# Smoking in pregnancy, cord blood cotinine and risk of celiac disease diagnosis in offspring

**DOI:** 10.1007/s10654-019-00522-5

**Published:** 2019-04-29

**Authors:** Karl Mårild, German Tapia, Øivind Midttun, Per M. Ueland, Maria C. Magnus, Marian Rewers, Lars C. Stene, Ketil Størdal

**Affiliations:** 1 Division for Mental and Physical Health, Norwegian Institute of Public Health, Oslo, Norway; 2 Department of Pediatrics, The Sahlgrenska Academy, University of Gothenburg, Gothenburg, Sweden; 3 Bevital AS, Bergen, Norway; 4 Department of Clinical Science, University of Bergen, Bergen, Norway; 5 Laboratory of Clinical Biochemistry, Haukeland University Hospital, Bergen, Norway; 6 MRC Integrative Epidemiology Unit, University of Bristol, Bristol, UK; 7 Department of Population Health Sciences, Bristol Medical School, Bristol, UK; 8 Barbara Davis Center, University of Colorado, Aurora, CO USA; 9 Department of Pediatrics, Østfold Hospital Trust, Grålum, Norway; 10 Department of Pediatrics, Queen Silvia Children’s Hospital, 41678 Gothenburg, Sweden

**Keywords:** Celiac disease, Cohort studies, Environmental tobacco smoke, Human leukocyte antigen, Smoking cessation, Registries

## Abstract

Ecological observations suggest an inverse relationship between smoking in pregnancy and celiac disease (CD) in offspring. While individual-level analyses have been inconsistent, they have mostly lacked statistical power or refined assessments of exposure. To examine the association between pregnancy-related smoking and CD in the offspring, as well as its consistency across data sets, we analyzed: (1) The Norwegian Mother and Child Cohort (MoBa) of 94,019 children, followed from birth (2000–2009) through 2016, with 1035 developing CD; (2) a subsample from MoBa (381 with CD and 529 controls) with biomarkers; and (3) a register-based cohort of 536,861 Norwegian children, followed from birth (2004–2012) through 2014, with 1919 developing CD. Smoking behaviors were obtained from pregnancy questionnaires and antenatal visits, or, in the MoBa-subsample, defined by measurement of cord blood cotinine. CD and potential confounders were identified through nationwide registers and comprehensive parental questionnaires. Sustained smoking during pregnancy, both self-reported and cotinine-determined, was inversely associated with CD in MoBa (multivariable-adjusted [a] OR = 0.61 [95%CI, 0.46–0.82] and aOR = 0.55 [95%CI, 0.31–0.98], respectively); an inverse association was also found with the intensity of smoking. These findings differed from those of our register-based cohort, which revealed no association with sustained smoking during pregnancy (aOR = 0.97 [95%CI, 0.80–1.18]). In MoBa, neither maternal smoking before or after pregnancy, nor maternal or paternal smoking in only early pregnancy predicted CD. In a carefully followed pregnancy cohort, a more-detailed smoking assessment than oft-used register-based data, revealed that sustained smoking during pregnancy, rather than any smoking exposure, predicts decreased likelihood of childhood-diagnosed CD.

**Electronic supplementary material:**

The online version of this article (10.1007/s10654-019-00522-5) contains supplementary material, which is available to authorized users.

## Introduction

Celiac disease (CD) is an autoimmune small-intestinal disorder prevalent in 1–3% of children in Western populations [1, 2]. In CD, genetic and environmental factors interplay in causing a loss of immune tolerance to gluten [3]. Human leukocyte antigens (HLA) are the primary genetic determinants of CD. Besides gluten ingestion, little is known about environmental triggers of CD.

Smoking in pregnancy has a profound impact on fetal development, including impaired growth and a multitude of effects on the immune system [4–6]. In recent decades, diminishing smoking rates among pregnant women have been observed in many countries [7], while the seroprevalence of CD has doubled [8, 9]. However, this inverse ecological relationship has not been supported by individual-level data which have shown a positive or no association, with one exception [10] (previous studies are summarized in Supplementary Table 1) [11–16].

This inconsistency of previous research could be owing to methodological restrictions, such as crude exposure assessments (e.g., “smoking/non-smoking”) which do not differ based on the intensity and duration of smoking in pregnancy [12–16]. No previous CD study has investigated the risk in relation to cotinine measurements [17], a biomarker of nicotine exposure; such an assessment could be important as women may underreport smoking in pregnancy. In 2014, our research group found a marked decreased risk of CD in children whose mothers smoked in pregnancy [10]. However, that study was restricted to self-reported smoking and had fewer means to assess the potential of confounding. Unraveling the link between intrauterine smoking exposure and childhood CD is important as it may hold clues to the pathogenesis of the disease.

The objective of this study was to examine if maternal smoking in pregnancy (measured by self-report and cord blood cotinine levels) was associated with CD diagnosis in her child. Secondary analyses considered paternal smoking and maternal smoking before or after pregnancy, which helps to evaluate potential confounding effects from family characteristics.

## Methods

**Fig. 1:**
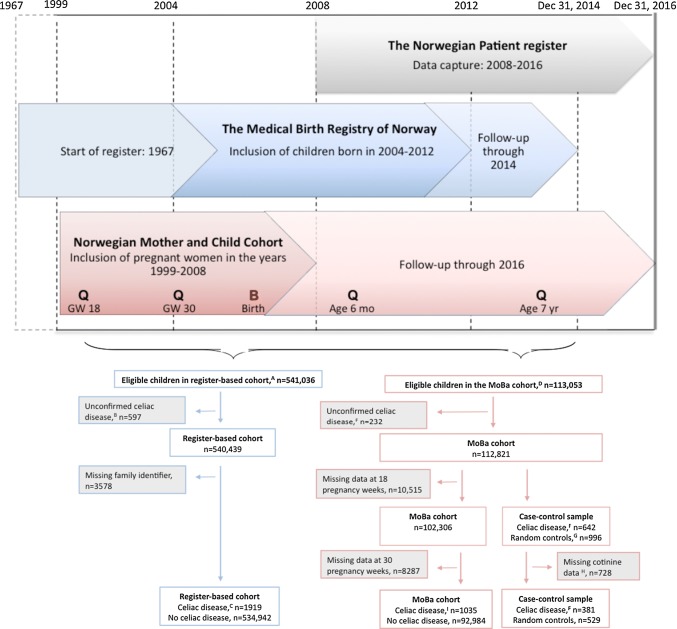
Study design and formation of study samples. In the Norwegian Mother and Child Cohort (MoBa) follow-up was conducted with parental questionnaires (denoted “Q”) at 18 and 30 gestational weeks (GW) and at child’s age 6 months and 7 years. Samples of cord blood (denoted “B”) were collected at birth. ^A^Norwegian children born in 2004–2012 and who survived the first 6 months of life. ^B^A single record of celiac disease (CD) in the Norwegian Patient Register (NPR). ^C^Minimum two entries of CD in NPR by December 31, 2014. ^D^Children who survived the first year of life. ^E^A single record of CD in NPR (without questionnaire confirmation). ^F^Minimum two records of CD in NPR by December 31, 2013, or confirmed through parental questionnaires. ^G^Among 113,053 children in MoBa we randomly selected 1009 controls; out of these, 11 children had CD and were reclassified as cases and two were excluded due to an unconfirmed CD diagnosis. ^H^Missing cord blood samples or exposed to moist snuff in pregnancy. ^I^Minimum two records of CD in NPR by December 31, 2016, or confirmed through parental questionnaires. Out of 1035 children with CD, 18 (1.7%) were identified solely based on parent reports and had no record of CD in the NPR (an additional 9 children who were included based on parent reports had a single entry of CD in the NPR)

This study is part of the Norwegian Mother and Child Cohort Study (MoBa), a population-based pregnancy cohort study [18]. Pregnant women were recruited across Norway in the years 1999–2008, and 41% of eligible women participated. The present study used prospective parent-reported smoking data on 94,019 children in MoBa and measurements of cord blood cotinine for a case–control sample nested within the cohort. In a separate analysis we assessed maternal smoking in a register-based cohort of 536,861 Norwegian children (Fig. 1, flowchart).

### Study samples

#### MoBa pregnancy cohort and nested case–control sample

Out of a cohort of 94,019 children in MoBa, 1035 children (1.1%) were diagnosed with CD by December 31, 2016. Nested within MoBa we designed a case–control study using stored cord blood samples from 381 children with CD diagnosed by the end of 2013 and 529 randomly selected controls without the disease (Fig. 1). We defined CD as minimum two records of the International Classification of Diseases (ICD)-10 code K90.0 in the Norwegian Patient Register (NPR) or CD reported by parental questionnaires administered at child’s age 7 years. Since January 1st, 2008, the NPR contains individual-level diagnostic data on virtually all inpatient and hospital-based outpatient care in Norway. We have previously described the identification of CD using the NPR [19]. While this register will identify children diagnosed prior to 2008 (start of NPR) provided they are followed as recommended in the outpatient clinic, data on parent reported CD enabled us to identify MoBa-children without such a follow-up. Out of 1035 MoBa-children with CD, 18 (1.7%) were identified solely based on parent reports and had no record of CD in the NPR (an additional 9 children who were included based on parent reports had a single entry of CD in the NPR).

We choose to define CD as at least two records of the disease in NPR in order to rule out children who during work up for the disease receive a preliminary working diagnosis of CD while waiting for histological or serological confirmation of the disease. We have previously validated CD in the MoBa cohort and found that > 92% of the diagnoses were confirmed by the parents [20].

#### Register-based cohort

Using the Medical Birth Registry of Norway [21] we identified children born between 2004 and 2012 (n = 536,861; Fig. 1). Out of these, 1919 children (0.4%) were diagnosed with CD by December 31, 2014, as defined by repeated ICD-10 codes of CD in the NPR. This register-based analysis was carried out in order to facilitate the comparison of our results with those of previous register-based studies [12–15]. However, these data were collected independently of MoBa resulting in a different follow-up length across cohorts. Data on parent reports of CD were not available in our register-based cohort.

### Exposures

#### MoBa pregnancy cohort: questionnaire data on parental smoking

Data on smoking were collected from questionnaires administered around weeks 18 and 30 of pregnancy (covering the period before pregnancy through pregnancy week 17 and pregnancy weeks 18–30, respectively) and at child’s age 6 months (smoking after pregnancy week 30 and up until child’s age 6 months). Mothers reported ever-smoking, smoking in the last 3 months before pregnancy, smoking duration in pregnancy and her average daily number of cigarettes smoked. The mother also described the father’s smoking habits as smoking/non-smoking before or during pregnancy, respectively. Prenatal exposure to environmental tobacco smoke (ETS) was quantified by the average number of hours per day of pregnancy the mother was exposed to secondhand smoke. Postnatal ETS exposure was quantified by the average sum of daily cigarettes smoked by the mother and father in the child’s first 6 months of life [22]. Excerpts of questionnaires are available as Supplementary Material.

To reduce the impact of erroneously registered data, discrepancies in smoking exposure as defined by different questionnaire items were treated as missing. The mothers of 191 children had inconsistencies regarding never-smoking status or pregnancy-related smoking; information on paternal smoking in pregnancy, as reported by the mother, was inconsistent for 5290 children. Never-smoking mothers and those not smoking in early pregnancy were assumed continuing as nonsmokers in later pregnancy when such data were missing. The primary exposure was maternal smoking in pregnancy. To aid causal inference, paternal smoking and maternal smoking before or after pregnancy were examined as negative controls.

#### Nested case–control sample: cotinine in cord blood

For a case–control sample nested within MoBa, we retrieved stored umbilical-cord plasma for measurements of cotinine, a nicotine metabolite, which as detected in cord blood is considered the gold standard of measuring fetal tobacco exposure at the end of pregnancy [17]. As previously described [23], cord blood was collected in EDTA tubes and shipped to the MoBa Biobank where plasma was separated before storage at − 80 °C. Samples were then thawed and, blinded to case–control status, shipped in one batch to the Bevital laboratory (Bergen, Norway) where cotinine was measured using liquid chromatography-tandem mass spectrometry [24]. Based on previous literature [25], we defined children with plasma cotinine ≥ 30 nmol/L as being exposed to daily maternal smoking in pregnancy. Children with cotinine concentrations ranging from 1.0 nmol/L (limit of detection) through 29 nmol/L were regarded as exposed to ETS or to occasional maternal smoking and children with undetectable cotinine concentrations were defined as not exposed to smoke.

#### Register-based cohort: maternal smoking recorded in the Medical Birth Registry of Norway

Information on maternal smoking (current non-smoker, occasional and daily smoker) was collected and recorded in maternal pregnancy records around 10 and 36 weeks of pregnancy as part of the universally accessible antenatal care [26]. Mothers may decline to register smoking status. The smoking data in the Medical Birth Registry of Norway have been extensively studied and demonstrated expected associations with multiple outcomes [26–30].

### Other variables

Based on previous literature and available data we preselected adjustment variables of maternal, paternal and child characteristics that may affect a possible association between pregnancy-related smoking and CD diagnosis in offspring (see directed acyclic graph [Supplementary Figure 1]) [31–34]. Variables were categorized as shown in Tables 1, 2 and Supplementary Table 2. For each child, we retrieved sex, birth weight, gestational age, delivery mode, parental age at delivery, maternal diabetes and parity from the Medical Birth Registry of Norway and maternal education level as recorded by Statistics Norway. For MoBa-children pregnancy questionnaires provided data on parental income, occupation and cohabitation [35–37], paternal education level and maternal infections in pregnancy [20], while the child’s infection frequency by age 6 months and breastfeeding duration were collected from the 6-month questionnaire [32, 34]. Data on parental CD and type 1 diabetes were retrieved from the NPR (ICD-10 codes K90.0 and E10, respectively). Based on previous literature [38], we did not consider age at infant gluten introduction as a predictor for CD. For our analysis on cotinine we used information on the degree of visual hemolysis of all cord blood samples (see our previous publication for details [39]).

#### Genotyping for celiac disease-associated risk markers

In our nested case–control analysis of cord blood cotinine we accounted for the child’s HLA genotype classified as conferring a high risk for CD (DQ2.5/DQ2.5 or DQ2.5/DQ2.2), moderate risk (DQ2.5/X or at least one of DQ2.2 or DQ8) and a low risk (any other genotype) [40]. We computed a non-HLA genetic risk score for CD defined as the child’s sum of 44 risk alleles that previously have been linked to CD at genome wide significance [40–42]. Genotyping details are available as Supplementary Material.

### Statistical analyses

To reduce the impact of missing data on our results we conducted multiple imputation by chained equation [43, 44], imputing 25 datasets. Multiple imputation replaces missing values with plausible numbers derived from distributions of and relationships among observed variables in the data; the subsequent analyses thus account for some of the uncertainty of the missing data. We imputed missing information on all covariates and exposure variables in MoBa and the register-based cohort (range of missingness, 0.1–19%; Supplementary Tables 2 and 3). Our imputation model included the child’s CD status and selected covariates. Imputation methods consisted of predictive mean matching and logistic models, as appropriate. The results presented are from the multiple imputation analyses. Complete-case analyses, which ignore observations with incomplete covariates, are provided in Supplementary Figures 2 and 3.

We estimated odds ratios (ORs) for CD using logistic regression with robust variance estimates allowing for cluster-correlated data among siblings [45]. We primarily examined the association between maternal smoking in pregnancy, defined by questionnaire data, register-based data or cotinine cut-offs, and offspring CD. Because the fetal susceptibility to smoke changes during the course of pregnancy [46], we examined the CD risk according to exposure only in early pregnancy (≤ 17 pregnancy weeks) or in early and late pregnancy (≥ 18 pregnancy weeks, henceforth referred to as sustained smoking during pregnancy). The mother’s average number of cigarettes per day of pregnancy was analyzed as continuous and trichotomous variables (0 [no use]; 1–2; > 2 cigarettes per day). Secondary analyses considered paternal smoking and maternal smoking before or after pregnancy. We performed explorative analyses in MoBa examining the effect of timing of maternal smoking cessation and using smoking data recorded in the Medical Birth Registry of Norway. To test if any differences found in results across cohorts may be explained by the inclusion of parent reported CD in MoBa we performed a sensitivity analysis excluding 27 children with CD identified through parent reports.

All analyses were adjusted for calendar year of birth (model I) because of its negative confounding effect on our results; younger children were less likely to have developed CD and, related to secular trends [7], less likely to have been exposed to smoking in pregnancy. In model II we further adjusted for maternal education level. We considered model II to be our primary adjustment model because it contained covariates available in both MoBa and the register-based cohort. In MoBa and our nested case–control sample we were also able to adjust for parental type 1 diabetes, CD, income, occupation, cohabitation and paternal education level (model III). The covariates in model I–III were all considered to be important confounders. A sensitivity analysis considered the effect of additional adjustment for mediators (breastfeeding duration, delivery mode, gestational age and birth weight) and covariates which effect on CD where considered to be small (parity, parental age at delivery, maternal infections in pregnancy, the child’s infection frequency by age 6 months) [20, 31]. This sensitivity analysis also adjusted for offspring sex. The cotinine analysis included the degree of hemolysis of cord blood samples as a covariate in models I–III. Finally, we re-ran the analyses on cotinine adjusting for HLA and non-HLA genetic risk markers for CD. We used Stata 15 (StataCorp, TX) for statistical analyses.

### Ethics

This study was approved by the Regional Committee for Medical and Health Research Ethics of southeast Norway (reference numbers: 2013/144 and 2013/2114). Informed consent was obtained from all individual participants included in the study.

## Results

### MoBa pregnancy cohort: questionnaire data on parental smoking

The mothers of 19,773 children (21.0%) reported any smoking during pregnancy. Of these, 12,272 quit smoking before 18 pregnancy weeks while 7501 continued smoking beyond that period. Information independently extracted from the Medical Birth Registry of Norway showed that the mothers of 10,135 (10.8%) children were smoking around 10 weeks of pregnancy. Mothers who smoked were younger, less often employed and had a lower education level compared with non-smokers (Table 1).

**Table 1:** Characteristics of children in the Norwegian Mother and Child Cohort Study (MoBa)

	All	Maternal smoking in pregnancy		
		No smoking	Smoking < 18 preg. weeks	Smoking ≥ 18 preg. weeks
n (%)	94,019 (100)	74,246 (79.0)	12,272 (13.1)	7501 (8.0)
Celiac disease, n (%)	1035 (1.1)	851 (1.1)	132 (1.1)	52 (0.7)
Calendar year of birth, median (range)	2005 (2000–2009)	2005 (2000–2009)	2005 (2000–2009)	2004 (2000–2009)
Girls, n (%)	45,879 (48.8)	36,242 (48.8)	5960 (48.6)	3677 (49.0)
Birth weight (gram), median (IQR)	3600 (3250–3940)	3600 (3260–3940)	3635 (3290–3990)	3498 (3130–3830)
Gestational age (weeks), median (IQR)	40 (39–41)	40 (39–41)	40 (39–41)	40 (39–41)
Cesarean delivery, n (%)	13,903 (14.8)	10,643 (14.3)	2018 (16.4)	1242 (16.6)
Duration of full breastfeeding, n (%)				
< 3 months	31,987 (34.0)	23,682 (31.9)	4872 (39.7)	3433 (45.8)
3–5 months	27,243 (29.0)	20,814 (28.0)	3886 (31.7)	2543 (33.9)
≥ 5 months	34,789 (37.0)	29,750 (40.1)	3514 (28.6)	1524 (20.3)
Child’s infections age 0–6 months, median (IQR)	1 (0–2)	1 (0–2)	1 (0–2)	1 (0–2)
Parity, n (%)				
0 [first child]	42,708 (45.4)	32,654 (44.0)	6749 (55.0)	3305 (44.1)
1	33,255 (35.4)	26,989 (36.4)	3741 (30.5)	2525 (33.7)
≥ 2	18,056 (19.2)	14,603 (19.7)	1782 (14.5)	1671 (22.3)
Maternal age at delivery, median (IQR)	30 (27–33)	31 (28–33)	29 (26–32)	29 (25–33)
Maternal education level, n (%)				
≤ 9 years	7074 (7.5)	3777 (5.1)	1387 (11.3)	1910 (25.5)
10–12 years	27,541 (29.3)	19,203 (25.9)	4780 (39.0)	3557 (47.4)
≥ 13 years	59,404 (63.2)	51,266 (69.0)	6105 (49.7)	2034 (27.1)
Maternal occupation, n (%)				
Sick leave/studying	7690 (8.2)	5350 (7.2)	1296 (10.6)	1044 (13.9)
Unemployed	5854 (6.2)	4221 (5.7)	716 (5.8)	916 (12.2)
Employed	80,475 (85.6)	64,674 (87.1)	10,260 (83.6)	5541 (73.9)
Maternal annual income (NOK), n (%)				
< 200,000	27,686 (29.4)	19,718 (26.6)	4300 (35.0)	3669 (48.9)
200,000–399,999	55,425 (59.0)	45,031 (60.7)	6964 (56.8)	3429 (45.7)
≥ 400,000	10,908 (11.6)	9497 (12.8)	1008 (8.2)	403 (5.4)
Maternal type 1 diabetes, n (%)	528 (0.6)	400 (0.5)	70 (0.6)	58 (0.8)
Maternal celiac disease, n (%)	1002 (1.1)	827 (1.1)	118 (1.0)	57 (0.8)
Maternal infections in pregnancy, median (IQR)	1 (0–2)	1 (0–2)	1 (0–2)	1 (0–2)
Paternal age at delivery, n (%)				
≤ 29 years	25,563 (27.2)	18,494 (24.9)	4333 (35.3)	2737 (36.5)
30–34 years	36,565 (38.9)	29,693 (40.0)	4499 (36.7)	2373 (31.6)
≥ 35 years	31,890 (33.9)	26,060 (35.1)	3440 (28.0)	2391 (31.9)
Paternal education level, n (%)				
≤ 9 years	10,059 (10.7)	6137 (8.3)	1893 (15.4)	2029 (27.0)
10–12 years	38,009 (40.4)	28,033 (37.8)	5230 (48.3)	4045 (53.9)
≥ 13 years	45,951 (48.9)	40,075 (54.0)	4449 (36.3)	1427 (19.0)
Paternal occupation, n (%)				
Sick leave/studying	5142 (5.5)	3732 (5.0)	806 (6.6)	604 (8.1)
Unemployed	1710 (1.8)	1086 (1.5)	274 (2.2)	350 (4.7)
Employed	87,167 (92.7)	69,428 (93.5)	11,192 (91.1)	6547 (87.3)
Paternal annual income (NOK), n (%)				
< 200,000	10,619 (11.3)	7423 (10.0)	1730 (14.1)	1465 (19.5)
200,000–399,999	52,250 (55.6)	40,477 (54.5)	7170 (58.4)	4602 (61.4)
≥ 400,000	31,150 (33.1)	26,345 (35.5)	3371 (27.5)	1433 (19.1)
Paternal type 1 diabetes, n (%)	609 (0.6)	473 (0.6)	87 (0.7)	49 (0.7)
Paternal celiac disease, n (%)	312 (0.3)	260 (0.3)	28 (0.2)	24 (0.3)
Paternal smoking in pregnancy, n (%)	20,689 (22.0)	10,499 (14.1)	5384 (43.9)	4804 (64.0)
Parental cohabitation, n (%)	90,933 (96.7)	72,597 (97.8)	11,626 (94.7)	6710 (89.4)

Rounded average cell counts and percentages are shown based on results from imputed data

Data from MoBa questionnaires, the Norwegian Patient Registry and the Medical Birth Registry of Norway

*IQR*, interquartile range; *NOK*, Norwegian krone, the national currency of Norway; *SD*, standard deviation

**Table 2:** Association of cord blood cotinine concentration and childhood celiac disease

	Celiac disease n = 381 (%)	Random controls n = 529 (%)	Model I		Model II		Model III	
			aOR	95% CI	aOR	95% CI	aOR	95% CI
Trichotomous cotinine groups [“Maternal exposure level”]								
*Cotinine* < *1.0* *nmol/L* [“No smoke exposure”]	329 (86.4)	433 (81.9)		Ref.		Ref.		Ref.
*Cotinine 1.0*–*29.9* *nmol/L* [“ETS/occasional smoking”]	32 (8.4)	49 (9.3)	0.84	0.53–1.33	0.84	0.53–1.35	0.83	0.51–1.36
*Cotinine *≥ *30.0* *nmol/L* [“Daily smoking”]	20 (5.2)	47 (8.9)	0.54	0.31–0.95	0.55	0.31–0.98	0.60	0.33–1.10
Per change in cotinine group^A^			0.76	0.59–0.97	0.77	0.60–0.98	0.79	0.60–1.03

^A^Analysis of trend over categories of increasing cotinine concentrations

All analyses were adjusted for calendar year of birth and degree of hemolysis of cord blood samples (model I). Model II also included maternal education level, while model III also accounted for parental type 1 diabetes, celiac disease, income, occupation, cohabitation and paternal education level. Missing information on covariates in models II–III imputed by chained Eqs.

*95% CI*, 95% confidence interval; *aOR*, adjusted odds ratio; *ETS*, environmental tobacco smoke

**Fig. 2:**
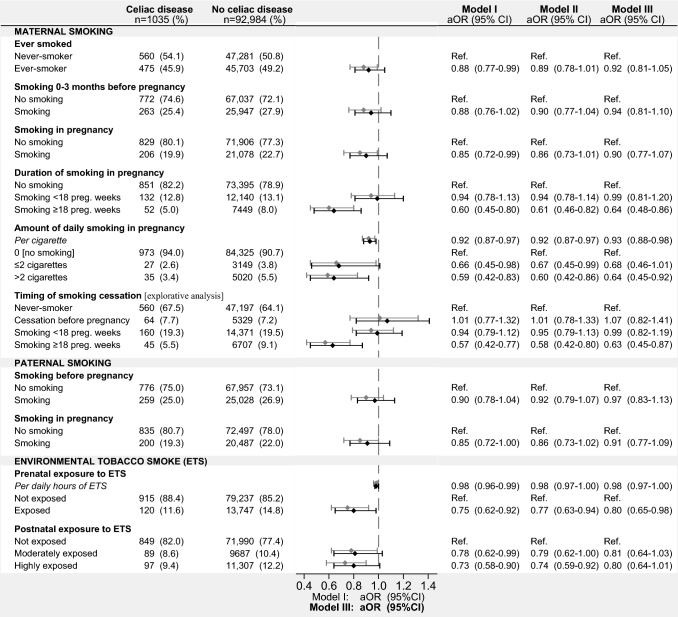
Association of parental smoking and childhood celiac disease in the MoBa cohort. Missing covariate and exposure information imputed by chained equations. All analyses were adjusted for calendar year of birth (model I). Model II also included maternal education level, while model III in addition to previous covariates also accounted for parental type 1 diabetes, celiac disease, income, occupation, cohabitation and paternal education level. Explorative analysis based on 74,433 children with data on timing of maternal smoking cessation. Inconsistent smoking exposure were treated as missing; the mothers of 191 children had inconsistencies regarding never-smoking status or pregnancy-related smoking; information on paternal smoking in pregnancy, as reported by the mother, was inconsistent for 5290 children. *95% CI*, 95% confidence interval; *aOR*, adjusted odds ratio

Maternal smoking beyond 18 weeks of pregnancy, but not a shorter duration of pregnancy-related smoking, was associated with a 40% reduction in risk of CD diagnosis in offspring (Fig. 2 [model I]). Results remained essentially unchanged when excluding 27 children with CD who were identified through parent reports of CD (data not shown). An inverse association was also found with the intensity of smoking in pregnancy. These associations remained largely unchanged when adjusting for socioeconomic characteristics (Fig. 2 [model III]) and for additional characteristics linked to smoking and CD diagnosis (Supplementary Figure 4). In contrast, neither paternal smoking nor maternal smoking before or after pregnancy was associated with offspring CD when adjusted for socioeconomic characteristics (Fig. 2).

### Nested case–control sample: cotinine in cord blood

Characteristics of the nested case–control sample were largely similar to those of the entire MoBa cohort, with the exception of a lower cesarean delivery rate (Supplementary Table 4). As defined by detectable cord blood cotinine concentrations (≥ 1.0 nmol/L), 18.1% (96/529) of randomly selected controls were exposed to smoking at the end of pregnancy. Notably, in about one-third of these children, neither parental smoking nor ETS exposure around the time of birth were reported in data collected at child’s age 6 months.

Adjusting for birth year, the OR for CD was 0.54 (95% CI, 0.31–0.95) for cotinine-determined daily smoking and 0.84 (95% CI, 0.53–1.33) for occasional smoking/ETS exposure at the end of pregnancy, compared with children with undetectable cotinine concentrations (“unexposed to smoke”). Adjusting for socioeconomic characteristics left ORs largely unchanged, albeit with wider CIs (Table 2). A similar pattern of results was found in analyses accounting for CD-associated genetic risk markers (Supplementary Table 5).

### Register-based cohort: maternal smoking recorded in the Medical Birth Registry of Norway

**Fig. 3:**
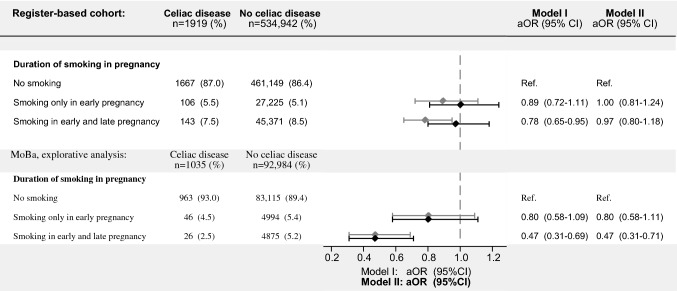
Association of maternal smoking in pregnancy recorded in the Medical Birth Registry of Norway and celiac disease diagnosis in offspring. Smoking recorded around 10 and 36 weeks of pregnancy. The primary analysis was based on a register-based cohort of Norwegian children born in 2004–2012. The explorative analysis examines the association among participants of the Norwegian Mother and Child (MoBa) cohort. Missing covariate and exposure information imputed by chained equations. Model I was adjusted for calendar year of birth while model II was further adjusted for maternal education level. Children with inconsistent data on smoking exposure (n = 1199) were excluded from the analysis. *95% CI*, 95% confidence interval; *aOR*, adjusted odds ratio

Compared with MoBa, the register-based cohort had a lower maternal education level (Table 3). The mothers of 72,846 (13.6%) children were smoking around 10 weeks of pregnancy, which was higher compared with the 10.8% maternal smoking rate in MoBa at that stage of pregnancy. Adjusting for birth year, children whose mother currently smoked around 10 or 36 pregnancy weeks had an OR for CD of 0.89 (95% CI, 0.72–1.11) and 0.78 (95% CI, 0.65–0.95), respectively, compared with children of non-smoking mothers. The inverse association of sustained smoking during pregnancy was completely removed when adjusting for maternal education level, in contrast to MoBa where we found unchanged association pattern before and after adjusting for maternal education (Fig. 3).

**Table 3:** Characteristics of register-based cohort

	All	Maternal smoking in pregnancy^A^			
		No smoking	Smoking 10 preg. weeks	Smoking 36 preg. weeks	Inconsistent data^B^
n (%)	536,861 (100)	462,816 (86.2)	27,331 (5.1)	45,514 (8.5)	1199 (0.2)
Celiac disease, n (%)	1919 (0.4)	1667 (0.4)	106 (0.4)	143 (0.3)	4 (0.3)
Calendar year of birth, median (IQR)	2008 (2004–2012)	2008 (2004–2012)	2007 (2004–2012)	2008 (2004–2012)	2008 (2004–2012)
Girls, n (%)	261,281 (48.7)	225,263 (48.7)	13,317 (48.7)	22,137 (48.6)	564 (47.1)
Birth weight (g), median (IQR)	3530 (3180–3880)	3550 (3200–3890)	3540 (3180–3890)	3355 (3000–3700)	3460 (3110–3800)
Gestational age (weeks), median (IQR)	40 (39–41)	40 (39–41)	40 (39–41)	39 (38–40)	40 (38–40)
Cesarean delivery, n (%)	90,021 (16.8)	76,993 (16.6)	4904 (17.9)	7905 (17.4)	219 (18.3)
Parity, n (%)					
0 [first child]	226,246 (42.1)	192,051 (41.5)	15,416 (56.4)	18,323 (40.3)	455 (38.0)
1	191,690 (35.7)	168,788 (36.5)	7705 (28.2)	14,751 (32.4)	447 (37.3)
≥ 2	118,924 (22.2)	101,977 (22.0)	4210 (15.4)	12,440 (27.3)	297 (24.8)
Maternal age at delivery, median (IQR)	30 (26–33)	30 (27–34)	27 (23–32)	28 (23–32)	29 (25–33)
Maternal education level, n (%)					
≤ 9 years	92,526 (17.2)	63,393 (13.7)	7767 (28.4)	20,989 (46.1)	377 (31.5)
10–12 years	160,207 (29.8)	130,971 (28.3)	10,960 (40.1)	17,818 (39.1)	459 (38.3)
≥ 13 years	284,127 (52.9)	268,453 (58.0)	8604 (31.5)	6707 (14.7)	363 (30.3)
Maternal diabetes, n (%)					
Pre-gestational	3990 (0.7)	3359 (0.7)	215 (0.8)	408 (0.9)	8 (0.7)
Gestational	8433 (1.6)	7304 (1.6)	434 (1.6)	672 (1.5)	22 (1.9)
Paternal age at delivery, median (IQR)	32 (29–37)	33 (29–37)	30 (26–35)	31 (26–36)	31 (27–36)

^A^Duration of smoking in pregnancy divided into mutually exclusive groups: no smoking, smoking up to 10 pregnancy weeks and continued smoking until 36 pregnancy weeks

^B^Children whose mothers started smoking in late pregnancy based on results from imputed data

Rounded average cell counts and percentages are shown based on results from imputed data

Data from the Norwegian Patient Registry and the Medical Birth Registry of Norway

*IQR* interquartile range

## Discussion

In Moba, self-reported and cotinine-determined sustained smoking during pregnancy, rather than any smoking exposure, were inversely associated with CD diagnosis. These findings differed from those of our register-based cohort, where we observed no associations with maternal smoking. These inconsistencies in results across closely related populations could reflect differences in the detail of smoking assessment, that if resolved may shed new light on intrauterine influences on CD development.

Similar to most previous studies we found no association between pregnancy-related smoking and offspring CD when confined to register-based data on smoking (previous studies are summarized in Supplementary Table 1) [12–15]. Generally, such observations are prone to measurement error by relying on dichotomized smoking assessments (e.g., “smoking/non-smoking”) at a single time-point (typically in early pregnancy). Consequently, few previous studies have been able to differentiate heavy from light smoking or early from late pregnancy smoking. Still other studies have relied on limited data on potential confounders [11, 12], leaving unanswered questions whether those modest relationships found were causal, confounded or a coincidence.

Hence, one explanation to our results my be that in using MoBa’s more detailed smoking assessments, including cotinine-determined smoking status, we were able to unmask a true inverse relationship between sustained smoking during pregnancy and childhood CD. This association persisted after carefully adjustments for a multitude of potential confounders. The lack of associations with paternal smoking, and maternal smoking before or after pregnancy, do not support residual confounding by health-affecting family characteristics as a sole cause of our results.

Assuming the observed association to be true, our results point towards a role of the intrauterine environment in CD etiology. Potential mechanisms warrant further study, but may involve programming of the fetal immune system or gut development, possibly via differential fetal DNA methylation [47]. A meta-analysis of the epigenetic effects of maternal smoking on cord blood suggests a possible influence on regulatory T-cells [48], which are instrumental in maintaining immune-tolerance. However, nicotine, and many other compounds of combustible tobacco products, may also directly affect the fetal immune system with implications for disease vulnerability later in life [4, 49]. Indeed, both experimental and observational studies have shown inverse associations between pregnancy-related smoking and childhood T1D [50–52], a disease that shares several etiological traits with CD [53].

We acknowledge that any potential beneficial effect of maternal smoking on offspring CD risk would not outweigh the harmful effects of smoking. However, future studies investigating the potential mechanisms underlying this association may lead to better understanding of the pathogenesis of CD which may, by other means than changing smoking advice, lead to methods of disease prevention.

Several non-causal explanations can be proposed to our finding of an inverse relationship of sustained smoking during pregnancy with CD diagnosed in MoBa, but not observed at the national level. As in any observational study we cannot rule of that residual or unmeasured confounders, such as childhood feeding practices [54], may have influenced our results. On the other hand, the potential effects of residual confounding may be less in the socially more homogeneous MoBa (Table 1, descriptive characteristics) as compared with those of our national register-based cohort (Table 3).

Two-thirds of the mothers smoking in early pregnancy, often before the pregnancy was confirmed, quit smoking during pregnancy. We do not know the extent to which these results can be generalized to other populations with different smoking habits or if sustained smoking during pregnancy may be related to confounding behavioral characteristics not captured by our adjustment models or by the use of negative controls (e.g. paternal smoking). The participation rate in MoBa was 41% which could influence the generalizability of those results. A previous comparison between mothers enrolling into MoBa and all Norwegian women giving birth at that time has shown that the cohort participants were older, less likely to have more than two previous births and more likely not to smoke during pregnancy (differences that were also revealed between MoBa and our register-based cohort) [55]. Such differences in prevalence estimates were not found to significantly influence exposure-outcome associations between participants in MoBa and the general population [55].

This study has several strengths, including its large sample sizes that gave precise relative risk estimates. The longitudinal data collection of smoking before CD was diagnosed in the child minimizes the risk of recall bias. We also took advantage of smoking data collected from independent sources, self-reported and defined by cord blood cotinine measurements, which increase the validity of our findings. That approach also allowed us in greater detail than previous works, to explore the effects of timing, intensity and duration of smoking and to disaggregate the effects of pre- and postnatal smoking exposure. Finally, using both questionnaire and register data we were able to comprehensively adjust for confounding socioeconomic characteristics.

Among the limitations of this study is that we were only able to ascertain diagnosed CD. Hence, we were unable to estimate the association of pregnancy-related smoking with subclinical, screening-detected CD. This is an important distinction because most children with CD will remain undiagnosed [56], and possibly more often so among those of low socioeconomic position [57]. Speculatively, a low-skilled mother, who is more likely to smoke, might also be more reluctant or less able to seek medical care and to request CD screening of her child. However, the lower CD prevalence in the register-based cohort (0.4%), compared with MoBa (1.1%), is likely foremost related to its shorter follow-up rather than lower ascertainment of CD (the CD prevalence in MoBa has increased with longer follow-up [10]). The coverage of CD diagnosis in NPR (launched in 2008) may in fact be higher for the register-based cohort (born in 2004–2012) than for the older cohort of children in MoBa (born in 2000–2009). Although, the effects of sustained smoking during pregnancy was consistent across subgroups of varying follow-up time in MoBa (data not shown) these differences in follow-up time, as well as in the used definitions of CD, make results not directly comparable across cohorts.

Notably, in one-third of children with detectable cord blood cotinine concentrations, neither parental smoking nor ETS exposure around the time of birth were reported. This inconsistency may not only reflect differences in the method used to define smoking exposure but also differences in terms of when such data were collected; while cotinine was measured in cord blood collected at birth, self-reported smoking data of that time were retrieved from questionnaires administered at child’s age 6 months. However, the consistency in our ORs for CD according to cotinine-defined and self-reported duration of smoking in MoBa argues against that selective missingness of reported smoking may have caused spurious results.

Finally, we also acknowledge that missing covariate and exposure information is a potential, but unverifiable, source of bias. To reduce the risk of such bias, we applied multiple imputation; this approach provides valid estimates given missingness in itself is unrelated to why observations are missing (i.e., missing at random).

In conclusion, sustained smoking during pregnancy, rather than any smoking exposure, was inversely associated with CD diagnosis in MoBa. Causality cannot be ascertained. However, in the context of detailed exposure assessments and comprehensive adjustment models these results may shed new light on intrauterine influences on CD development.

## Electronic supplementary material

Below is the link to the electronic supplementary material.

Supplementary material 1 (PDF 3300 kb)

## Abbreviations

aOR: Adjusted odds ratio
CD: Celiac disease
CI: Confidence interval
ETS: Environmental tobacco smoke
HLA: Human leukocyte antigen
ICD: International Classification of Diseases
MoBa: Norwegian Mother and Child Cohort Study
NPR: Norwegian Patient Register
OR: Odds ratio
SD: Standard deviation
