# Supplementary material for: Smoking in pregnancy, cord blood cotinine and risk of celiac disease diagnosis in offspring
Source: Eur J Epidemiol. 2019 Apr 29;34(7):637–49. doi: 10.1007/s10654-019-00522-5 (PMC6548867; doi:10.1007/s10654-019-00522-5)
Supplement: Supplementary file 1 — Supplementary material 1 (PDF 3300 kb) [file 10654_2019_522_MOESM1_ESM.pdf]

# Supplementary Material

SUPPLEMENTARY TABLES ..... 2
SUPPLEMENTARY TABLE 1. PREVIOUS LITERATURE ON THE ASSOCIATION BETWEEN PARENTAL SMOKING IN PREGNANCY AND CELIAC DISEASE (CD) IN OFFSPRING. ..... 2
SUPPLEMENTARY TABLE 2. CHARACTERISTICS OF CHILDREN IN THE NORWEGIAN MOTHER AND CHILD COHORT STUDY (MOBA). DESCRIPTIVE STATISTICS BASED ON COMPLETE-CASE DATA. ..... 5
SUPPLEMENTARY TABLE 3. CHARACTERISTICS OF CHILDREN IN REGISTER-BASED COHORT. DESCRIPTIVE STATISTICS BASED ON COMPLETE-CASE DATA. ..... 8
SUPPLEMENTARY TABLE 4. CHARACTERISTICS OF NESTED CASE-CONTROL SAMPLE ..... 10
SUPPLEMENTARY TABLE 5. COMPLETE-CASE ANALYSIS ON THE ASSOCIATION OF CORD BLOOD COTININE CONCENTRATION AND CHILDHOOD CELIAC DISEASE. ..... 12
SUPPLEMENTARY FIGURE LEGENDS ..... 13
SUPPLEMENTARY FIGURE 1. DIRECTED ACYCLIC GRAPH DEPICTING THE HYPOTHESIZED RELATIONSHIPS BETWEEN MATERNAL SMOKING IN PREGNANCY AND OFFSPRING CELIAC DISEASE. ..... 13
SUPPLEMENTARY FIGURE 2. COMPLETE-CASE ANALYSES OF THE ASSOCIATION OF PARENTAL SMOKING WITH CHILDHOOD CELIAC DISEASE IN THE MOBA COHORT. ..... 15
SUPPLEMENTARY FIGURE 3. COMPLETE-CASE ANALYSIS ON THE ASSOCIATION OF MATERNAL SMOKING IN PREGNANCY RECORDED IN THE MEDICAL BIRTH REGISTRY OF NORWAY AND CELIAC DISEASE DIAGNOSIS IN OFFSPRING. ..... 17
SUPPLEMENTARY FIGURE 4. SENSITIVITY ANALYSIS ON THE EFFECT OF ADDITIONAL ADJUSTMENTS OF PARENTAL AND CHILD CHARACTERISTICS RECORDED IN THE MOBA COHORT. ..... 18
SUPPLEMENTARY METHODS ..... 20
RECORDING OF MATERNAL SMOKING IN THE MEDICAL BIRTH REGISTRY OF NORWAY ..... 20
DETAILS ON GENOTYPING OF CORD BLOOD ..... 20
EXCERPTS FROM MOBA-QUESTIONNAIRES USED TO ASSESS PARENTAL SMOKING ..... 22

## SUPPLEMENTARY TABLES

***Supplementary Table 1.*** *Previous literature on the association between parental smoking in pregnancy and celiac disease (CD) in offspring.*

| **Authors** (year of publication) | **Country** | **Design** | **Participants** | **Data sources** | | **Selected covariates** | **Main findings** |
|---|---|---|---|---|---|---|---|
| | | | | *CD* n=number of children with CD | *Smoking* | | |
| **Adlercreutz *et al.*** (2014)[A, 1] | Sweden | National register-based study | 768,395 children born in 1987-1993 | The National Patient Register n=4008 | The Swedish MBR: mom's smoking/non-smoking at 6-12 GW | - | 25% of children with CD were exposed to maternal smoking in early pregnancy (1001/4008) as compared with 23% among those in the reference group (176,044/760,060) (OR 1.02; 95% CI 0.95-1.10). |
| **Aronsson *et al.*** (2015)[2] | Sweden, Finland, Germany, United states | Multinational birth cohort | 6436 children genetically at risk for CD | Repeated CD screening. n=773 with CD autoimmunity (consecutive positive tTG), of whom 307 had CD | Parental questionnaires completed at 3-4 months postpartum: mom's smoking/non-smoking any time in pregnancy | Presents unadjusted estimates. Genetic and dietary data are included in other parts of the study. | 63 out of 773 children with CD autoimmunity and 28 out of 307 children with CD were exposed to maternal smoking in pregnancy (CD autoimmunity: HR 0.85, 95% CI 0.65-1.09; CD: HR 0.98, 95% CI 0.67-1.45). |
| **Emilsson *et al.*** (2014)[B, 3] | Norway | National cohort | 108,478 children born in 2000-2009. Earlier version of the Norwegian Mother and Child Cohort Study. | Parental questionnaires and the Norwegian Patient Registry n=650 | Parental questionnaires administered at 18 GW and at 6 months postpartum: Mom's smoking/non-smoking at 18 GW; parental smoking/non-smoking pre-, post pregnancy | Maternal CD, type 1 diabetes, age at delivery, parity, education level, mode of delivery, birth weight, gestational age, child's attained age at end of study, breastfeeding duration, timing of gluten introduction. | 6% (35/617) of children later developing CD were exposed to maternal smoking at 18 pregnancy weeks, compared with 8% (8430/101,413) of children without CD, corresponding to an adjusted OR of 0.66 (95% CI 0.44-0.97). Maternal and paternal smoking in the last three months of pregnancy yielded an adjusted OR of 0.86 [95% CI 0.68-1.08] and 0.88 [95% CI 0.77-1.00], respectively). Maternal and paternal smoking at child's age 4-6 months was associated with an adjusted OR of 0.85 (95% CI 0.71-1.03) and 0.84 (95% CI 0.73-0.96), respectively. |

| | | | | | | | |
|---|---|---|---|---|---|---|---|
| **Ludvigsson *et al.*** (2005) [4] | Sweden | Regional cohort | 15,397 children born in 1997-1999. Part of the All Babies in Southeast Sweden Cohort | CD reported from regional pediatric departments. n=53 | Parental questionnaires administered at birth and at child's age one year: mom's smoking any time in pregnancy, her daily number of cigarettes, ETS exposure 0-12 months | Maternal education level, pre-pregnancy body mass index, breastfeeding duration | 17% of the children with CD were exposed to maternal smoking in pregnancy (9/53) as compared with 11% (1699/15,344) among children without CD (OR 1.64; 95% CI 0.80-3.37). Neither the number of cigarettes smoked during pregnancy nor maternal smoking in the child's first year of life was associated with childhood CD (both P-values>0.15). |
| **Namatovu *et al.*** (2016) [5] | Sweden | National register-based study | 1,912,204 children born in 1991-2009 | The National Swedish Childhood Celiac Disease Register n=6596 | The Swedish MBR: mom's smoking/non-smoking at 6-12 GW | Maternal age at delivery, salary, parity, infections in pregnancy, gestational age, delivery mode, birth weight | Maternal smoking in pregnancy was found among 14% of children with CD (853/6596) as compared with 13% (240,677/1,912,204) among children without CD (OR 1.0; 95% CI 0.9-1.1). |
| **Roberts *et al.*** (2009) [6] | England | Regional register-based study | 248,521 children born in 1970-1999 and included in the Oxford record linkage study database | CD diagnosed at inpatient or hospital-based day care admissions. n=90 | Data abstracted from maternity records in the years 1973-1999: mom's smoking any time in pregnancy | Maternal CD, martial-status, age at delivery, parity, maternal occupation, delivery mode, gestational age, breastfeeding (yes/no), birth weight, | In bivariate analysis the cumulative CD incidence per 100,000 births was 56 (95% CI 33-87) in children exposed to maternal smoking in pregnancy vs. 26 (95% CI 18-38) among unexposed (P-value 0.01; P-value per cigarette smoked in pregnancy, 0.11). In the multivariate model maternal smoking was not significantly associated with CD (no risk estimates were reported). |
| **Sandberg-Bennich *et al.*** (2002)[A, 7] | Sweden | National register-based study | 1,182,056 children born in 1987-1997 | The National Patient Register n=3392 | The Swedish MBR: mom's smoking/non-smoking at 6-12 GW | Maternal age at delivery, parity, year of birth | Maternal smoking in early pregnancy was significantly associated with offspring CD: OR 1.10; 95% CI 1.01–1.19 |
| **Wingren *et al.*** (2012)[A, 8] | Sweden | National register-based study | 681,954 children born in 1987-1993 | The National Patient Register n=2641 | The Swedish MBR: mom's smoking/non-smoking at 6-12 GW | Maternal age at delivery, year of birth, parity, neonatal comorbidity | Maternal smoking in early pregnancy was not significantly associated with offspring CD: Boys, HR 1.09; 95% CI 0.95–1.26; Girls, HR 1.06; 95% CI 0.95–1.19. |

The listed papers were identified using a PubMed search on August 27, 2017, for (celiac disease[Title] OR coeliac disease[Title]) AND (pregnancy[Title/Abstract] OR perinatal[Title/Abstract] OR infant[Title/Abstract] OR offspring[Title/Abstract]). [A] Studies with largely overlapping study samples and data sources on CD and smoking status. [B] Partly overlapping study sample and outcome definitions as of the current study, but with the following distinctions; The previous study was restricted to self-reported data on smoking during and after pregnancy as recorded in the Norwegian Mother and Child Cohort Study (MoBa); exposure to pre-pregnancy smoking and environmental tobacco smoke were not examined and there were no data on smoking from the Norwegian Medical Birth Registry or of cotinine-determined smoking status. Compared with the current work, the previous study had a shorter follow-up for CD (eight versus eleven years of average follow-up) identifying almost half the number of CD cases compared with the present study. 95% CI, 95% confidence interval; CD, celiac disease; ETS, environmental tobacco smoke; GW, gestational weeks; HR, hazard ratio; OR, odds ratio; MFR, Medical Birth Register; tTG, tissue transglutaminase antibodies.

1. Adlercreutz EH, Wingren CJ, Vincente RP, Merlo J, Agardh D. Perinatal risk factors increase the risk of being affected by both type 1 diabetes and coeliac disease. *Acta Paediatr.* Feb 2015;104(2):178-184.
2. Aronsson CA, Lee HS, Liu E, et al. Age at gluten introduction and risk of celiac disease. *Pediatrics.* Feb 2015;135(2):239-245.
3. Emilsson L, Magnus M, Størdal K. Perinatal Risk Factors for Development of Celiac Disease in Children, Based on the Prospective Norwegian Mother and Child Cohort Study. *Clin Gastroenterol Hepatol 2015; 13:921-7.*
4. Ludvigsson JF, Ludvigsson J. Parental smoking and risk of coeliac disease in offspring. *Scand J Gastroenterol.* Mar 2005;40(3):336-342.
5. Namatovu F, Olsson C, Lindkvist M, et al. Maternal and perinatal conditions and the risk of developing celiac disease during childhood. *BMC Pediatr.* 2016;16(1):77.
6. Roberts SE, Williams JG, Meddings D, Davidson R, Goldacre MJ. Perinatal risk factors and coeliac disease in children and young adults: a record linkage study. *Aliment Pharmacol Ther.* Jan 2009;29(2):222-231.
7. Sandberg-Bennich S, Dahlquist G, Källen B. Coeliac disease is associated with intrauterine growth and neonatal infections. *Acta Paediatr.* 2002;91(1):30-33.
8. Wingren CJ, Agardh D, Merlo J. Revisiting the risk of celiac disease in children born small for gestational age: a sibling design perspective. *Scand J Gastroenterol.* Jun 2012;47(6):632-639.

***Supplementary Table 2.*** *Characteristics of children in the Norwegian Mother and Child Cohort Study (MoBa). Descriptive statistics based on complete-case data.*

| | All | Maternal smoking in pregnancy | | | |
|---|---|---|---|---|---|
| **n (%)** | 94,019 (100) | *No smoking* 73,806 (79) | *Smoking <18 preg. weeks* 12,182 (13) | *Smoking ≥18 preg. weeks* 7453 (8) | *Missing data* 578 |
| **Celiac disease, n (%)** | 1035 (1.1) | 848 (1.1) | 132 (1.1) | 52 (0.7) | 3 (0.5) |
| Missing data, n | 0 | 0 | 0 | 0 | 0 |
| **Calendar year of birth, median (range)** | 2005 (2000-2009) | 2005 (2000-2009) | 2005 (2000-2009) | 2004 (2000-2009) | 2005 (2000-2009) |
| Missing data, n | 0 | 0 | 0 | 0 | 0 |
| **Girls, n (%)** | 45,879 (48.8) | 36,035 (48.8) | 5916 (48.6) | 3655 (49.0) | 273 (47.2) |
| Missing data, n | 0 | 0 | 0 | 0 | 0 |
| **Birth weight (gram), median (IQR)** | 3600 (3250-3940) | 3600 (3260-3940) | 3636 (3290-3990) | 3498 (3130-3830) | 3605 (3230-3930) |
| Missing data, n | 52 | 39 | 7 | 6 | 0 |
| **Gestational age (weeks), median (IQR)** | 40 (39-41) | 40 (39-41) | 40 (39-41) | 40 (39-41) | 40 (39-41) |
| Missing data, n | 404 | 325 | 49 | 24 | 6 |
| **Cesarean delivery, n (%)** | 13,903 (14.8) | 10,580 (14.3) | 2001 (16.4) | 1232 (16.5) | 90 (15.6) |
| Missing data, n | 0 | 0 | 0 | 0 | 0 |
| **Duration of full breastfeeding, n (%)** | | | | | |
| <3 months | 28,690 (33.8) | 21,396 (31.8) | 4268 (39.3) | 2863 (45.0) | 163 (33.1) |
| 3-5 months | 24,624 (29.0) | 18,752 (27.9) | 3476 (32.0) | 2238 (35.2) | 158 (32.1) |
| ≥5 months | 31,574 (37.2) | 27,033 (40.2) | 3111 (28.7) | 1259 (19.8) | 171 (34.8) |
| Missing data, n | 9131 | 6625 | 1327 | 1093 | 86 |
| **Child's infections age 0-6 months, median (IQR)** | 1 (0-2) | 1 (0-2) | 1 (0-2) | 1 (0-2) | 1 (0-3) |
| Missing data, n | 10,671 | 7696 | 1595 | 1277 | 103 |
| **Parity, n (%)** | | | | | |
| 0 [first child] | 42,708 (45.4) | 32,483 (44.0) | 6706 (55.0) | 3285 (44.1) | 234 (40.5) |
| 1 | 33,255 (35.4) | 26,815 (36.3) | 3706 (30.4) | 2507 (33.6) | 227 (39.3) |
| ≥2 | 18,056 (19.2) | 14,508 (19.7) | 1770 (14.5) | 1661 (22.3) | 117 (20.2) |
| Missing data, n | 0 | 0 | 0 | 0 | 0 |
| **Maternal age at delivery, median (IQR)** | 30 (27-33) | 31 (28-33) | 29 (26-32) | 29 (25-33) | 30 (27-33) |
| Missing data, n | 0 | 0 | 0 | 0 | 0 |
| **Maternal education level, n (%)** | | | | | |
| ≤9 years | 7023 (7.5) | 3714 (5.1) | 1372 (11.3) | 1884 (25.4) | 53 (9.5) |

| | | | | | |
|---|---|---|---|---|---|
| 10-12 years | 27,407 (29.3) | 18,982 (25.8) | 4725 (38.9) | 3511 (47.4) | 189 (33.9) |
| ≥13 years | 59,158 (63.3) | 50,788 (69.1) | 6046 (49.8) | 2009 (27.1) | 315 (56.6) |
| Missing data, n | 431 | 322 | 39 | 49 | 21 |
| **Maternal occupation, n (%)** | | | | | |
| Sick leave/Studying | 7459 (8.1) | 5159 (7.1) | 1249 (10.4) | 996 (13.7) | 55 (10.1) |
| Unemployed | 5685 (6.2) | 4079 (5.6) | 691 (5.8) | 880 (12.1) | 35 (6.4) |
| Employed | 79,056 (85.8) | 63,221 (87.3) | 10,013 (83.8) | 5369 (74.1) | 453 (83.4) |
| Missing data, n | 1819 | 1347 | 229 | 208 | 35 |
| **Maternal annual salary (NOK), n (%)** | | | | | |
| <200,000 | 26,428 (29.1) | 18,757 (26.3) | 4077 (34.6) | 3435 (48.4) | 159 (30.3) |
| 200,000-399,999 | 53,794 (59.2) | 43,493 (60.9) | 6720 (57.1) | 3269 (46.1) | 312 (59.5) |
| ≥400,000 | 10,613 (11.7) | 9196 (12.9) | 977 (8.3) | 387 (5.5) | 53 (10.1) |
| Missing data, n | 3184 | 2360 | 408 | 362 | 54 |
| **Maternal type 1 diabetes, n (%)** | 528 (0.6) | 398 (0.5) | 69 (0.6) | 58 (0.8) | 3 (0.5) |
| Missing data, n | 0 | 0 | 0 | 0 | 0 |
| **Maternal celiac disease, n (%)** | 1002 (1.1) | 817 (1.1) | 117 (1.0) | 57 (0.8) | 11 (1.9) |
| Missing data, n | 0 | 0 | 0 | 0 | 0 |
| **Maternal infections in pregnancy, median (IQR)** | 1 (0-2) | 1 (0-2) | 1 (0-2) | 1 (0-2) | 1 (0-2) |
| Missing data, n | 0 | 0 | 0 | 0 | 0 |
| **Paternal age at delivery, n (%)** | | | | | |
| ≤29 years | 25,491 (27.2) | 18,333 (24.9) | 4286 (35.3) | 2700 (36.5) | 172 (29.9) |
| 30-34 years | 36,494 (38.9) | 29,493 (40.0) | 4460 (36.7) | 2343 (31.6) | 198 (34.4) |
| ≥35 years | 31,792 (33.9) | 25,822 (35.1) | 3404 (28.0) | 2361 (31.9) | 205 (35.7) |
| Missing data, n | 242 | 158 | 32 | 49 | 3 |
| **Paternal education level, n (%)** | | | | | |
| ≤9 years | 9547 (10.5) | 5833 (8.1) | 1793 (15.2) | 1857 (26.5) | 64 (12.0) |
| 10-12 years | 36,701 (40.3) | 26,970 (37.6) | 5683 (48.3) | 3811 (54.4) | 237 (44.4) |
| ≥13 years | 44,781 (49.2) | 38,919 (54.3) | 4293 (36.5) | 1336 (19.1) | 233 (43.6) |
| Missing data, n | 2990 | 2084 | 413 | 449 | 44 |
| **Paternal occupation, n (%)** | | | | | |
| Sick leave/Studying | 4905 (5.3) | 3563 (4.9) | 760 (6.4) | 547 (7.7) | 35 (6.5) |
| Unemployed | 1623 (1.8) | 1033 (1.4) | 259 (2.2) | 320 (4.5) | 11 (2.1) |
| Employed | 85,111 (92.9) | 67,551 (93.6) | 10,839 (91.4) | 6231 (87.8) | 490 (91.4) |

| | | | | | |
|---|---|---|---|---|---|
| Missing data, n | 2380 | 1659 | 324 | 355 | 42 |
| **Paternal annual salary (NOK), n (%)** | | | | | |
| <200,000 | 9475 (10.8) | 6719 (9.7) | 1515 (13.5) | 1188 (18.2) | 53 (10.5) |
| 200,000-399,999 | 48,779 (55.7) | 37,856 (54.6) | 6566 (58.6) | 4074 (62.4) | 283 (56.0) |
| ≥400,000 | 29,353 (33.5) | 24,792 (35.7) | 3120 (27.9) | 1272 (19.5) | 169 (33.5) |
| Missing data, n | 6412 | 4439 | 981 | 919 | 73 |
| **Paternal type 1 diabetes, n (%)** | 609 (0.6) | 472 (0.6) | 87 (0.7) | 49 (0.7) | 1 (0.2) |
| Missing data, n | 0 | 0 | 0 | 0 | 0 |
| **Paternal celiac disease, n (%)** | 312 (0.3) | 257 (0.3) | 28 (0.2) | 24 (0.3) | 3 (0.5) |
| Missing data, n | 0 | 0 | 0 | 0 | 0 |
| **Paternal smoking in pregnancy, n (%)** | 18,915 (20.2) | 9874 (13.4) | 4299 (35.4) | 4612 (62.2) | 130 (48.3) |
| Missing data, n | 555 | 185 | 26 | 35 | 309 |
| **Parental cohabitation, n (%)** | 90,647 (96.7) | 71,968 (97.8) | 11,512 (94.8) | 6625 (89.4) | 542 (96.4) |
| Missing data, n | 302 | 206 | 34 | 46 | 16 |

Data from MoBa questionnaires, the Norwegian Patient Registry and the Medical Birth Registry of Norway. IQR, interquartile range; NOK, Norwegian krone, the national currency of Norway.

***Supplementary Table 3.*** *Characteristics of children in register-based cohort. Descriptive statistics based on complete-case data.*

| n (%) | All<br>536,861 (100) | Maternal smoking in pregnancy [A]<br>*No smoking*<br>384,807 (87) | <br>*Smoking 10 preg. weeks*<br>21,618 (5) | <br>*Smoking 36 preg. weeks*<br>35,868 (8) | <br>*Missing data*<br>94,568 |
|---|---|---|---|---|---|
| **Celiac disease, n (%)** | 1919 (0.4) | 1369 (0.4) | 82 (0.4) | 119 (0.3) | 349 (0.4) |
| Missing data, n | 0 | 0 | 0 | 0 | 0 |
| **Calendar year of birth, median (range)** | 2008 (2004-2012) | 2008 (2004-2012) | 2007 (2004-2012) | 2008 (2004-2012) | 2008 (2004-2012) |
| Missing data, n | 0 | | | | 0 |
| **Girls, n (%)** | 261,281 (48.7) | 187,251 (48.7) | 10,509 (48.6) | 17,427 (48.6) | 46,094 (48.7) |
| Missing data, n | 0 | 0 | 0 | 0 | 0 |
| **Birth weight (gram), median (IQR)** | 3530 (3180-3880) | 3554 (3206-3900) | 3550 (3190-3900) | 3360 (3000-3710) | 3500 (3150-3850) |
| Missing data, n | 259 | 174 | 12 | 19 | 54 |
| **Gestational age (weeks), median (IQR)** | 40 (39-41) | 40 (39-41) | 40 (39-41) | 39 (38-40) | 40 (38-41) |
| Missing data, n | 3871 | 2675 | 192 | 362 | 642 |
| **Cesarean delivery, n (%)** | 90,021 (16.8) | 63,086 (16.4) | 3790 (17.5) | 6037 (16.8) | 17,108 (18.1) |
| Missing data, n | 0 | 0 | 0 | 0 | 0 |
| **Parity, n (%)** | | | | | |
| 0 [first child] | 226,233 (42.1) | 158,022 (41.1) | 12,201 (56.4) | 14,460 (40.3) | 41,550 (43.9) |
| 1 | 191,677 (35.7) | 140,844 (36.6) | 6116 (28.3) | 11,686 (32.6) | 33,031 (34.9) |
| ≥2 | 118,915 (22.2) | 85,916 (22.3) | 3300 (15.3) | 9719 (27.1) | 19,980 (21.1) |
| Missing data, n | 36 | 25 | 1 | 3 | 7 |
| **Maternal age at delivery, median (IQR)** | 30 (26-33) | 30 (26-33) | 27 (23-32) | 28 (23-32) | 30 (27-34) |
| Missing data, n | 19 | 10 | 0 | 0 | 9 |
| **Maternal education level, n (%)** | | | | | |
| ≤9 years | 87,780 (17.0) | 49,424 (13.4) | 5977 (28.1) | 15,812 (45.2) | 16,567 (18.6) |
| 10-12 years | 153,795 (29.8) | 106,859 (28.9) | 8669 (40.8) | 14,035 (40.1) | 24,232 (27.2) |
| ≥13 years | 273,684 (53.1) | 213,704 (57.8) | 6596 (31.1) | 5138 (14.7) | 48,246 (54.2) |
| Missing data, n | 21,602 | 14,820 | 376 | 883 | 5523 |
| **Maternal diabetes, n (%)** | | | | | |
| Pre-gestational | 3990 (0.7) | 2785 (0.7) | 166 (0.8) | 311 (0.9) | 728 (0.8) |
| Gestational | 8433 (1.6) | 6365 (1.7) | 352 (1.6) | 525 (1.5) | 1191 (1.3) |

| | | | | | |
|---|---|---|---|---|---|
| Missing data, n | 0 | 0 | 0 | 0 | 0 |
| **Paternal age at delivery, median (IQR)** | 32 (29-37) | 33 (29-37) | 30 (26-35) | 31 (26-36) | 33 (29-37) |
| Missing data, n | 6559 | 3992 | 318 | 827 | 1422 |

Data from the Norwegian Patient Registry and the Medical Birth Registry of Norway. [A] Duration of smoking in pregnancy divided into mutually exclusive groups: no smoking, smoking up to 10 pregnancy weeks and continued smoking until 36 pregnancy weeks. Missing data are largely due to a lack of consent to record smoking data in the Medical Birth Registry of Norway. IQR, interquartile range

***Supplementary Table 4.*** *Characteristics of nested case-control sample*

| n (%) | Nested case-control sample | |
|---|---|---|
| | *Celiac disease* n=381 | *Random controls* n=529 |
| **HLA genotype, n (%)** [A] | | |
| Other genotypes ("low risk for celiac disease") | 5 (1.4) | 159 (32.9) |
| DQ8 or DQ2 ("moderate risk") | 243 (68.8) | 306 (63.2) |
| DQ2.5/DQ2 ("high risk") | 105 (29.7) | 19 (3.9) |
| **No. of non-HLA risk alleles, median (IQR)** [B] | 44 (41-47) | 42 (39-45) |
| **Calendar year of birth, median (range)** | 2004 (2000-2009) | 2005 (2000-2009) |
| **Girls, n (%)** | 227 (59.6) | 260 (49.1) |
| **Birth weight (gram), median (IQR)** | 3570 (3280-3930) | 3590 (3275-3970) |
| **Gestational age (weeks), median (IQR)** | 40 (39-41) | 40 (39-41) |
| **Cesarean delivery, n (%)** | 43 (11.3) | 54 (10.2) |
| **Duration of full breastfeeding, n (%)** | | |
| <3 months | 86 (25.8) | 148 (33.1) |
| 3-5 months | 106 (31.8) | 138 (30.9) |
| ≥5 months | 141 (42.3) | 161 (36.0) |
| **Child's infections age 0-6 months, median (IQR)** | 1 (0-2) | 1 (0-2) |
| **Parity, n (%)** | | |
| 0 [first child] | 155 (40.7) | 233 (44.0) |
| 1 | 154 (40.4) | 195 (36.9) |
| ≥2 | 72 (18.9) | 101 (19.1) |
| **Maternal age at delivery, median (IQR)** | 30 (27-33) | 30 (27-33) |
| **Maternal education level, n (%)** | | |
| ≤9 years | 26 (7.1) | 33 (6.7) |
| 10-12 years | 108 (29.6) | 156 (31.8) |
| ≥13 years | 231 (63.3) | 302 (61.5) |
| **Maternal occupation, n (%)** | | |
| Sick leave/Studying | 34 (9.4) | 36 (7.3) |
| Unemployed | 20 (5.5) | 33 (6.7) |
| Employed | 307 (85.0) | 421 (85.9) |
| **Maternal annual salary (NOK), n (%)** | | |
| <200,000 | 109 (31.1) | 147 (30.7) |
| 200,000-399,999 | 214 (61.0) | 284 (59.3) |
| ≥400,000 | 28 (8.0) | 48 (10.0) |
| **Maternal type 1 diabetes, n (%)** | 5 (1.3) | 0 (0.0) |
| **Maternal celiac disease, n (%)** | 45 (11.8) | 2 (0.4) |
| **Maternal infections in pregnancy, median (IQR)** | 1 (0-2) | 1 (0-2) |
| **Paternal age at delivery, n (%)** | | |
| ≤29 years | 103 (27.1) | 157 (29.7) |
| 30-34 years | 158 (41.6) | 190 (36.0) |
| ≥35 years | 119 (31.3) | 181 (34.3) |
| **Paternal education level, n (%)** | | |
| ≤9 years | 33 (9.4) | 57 (11.9) |
| 10-12 years | 142 (40.3) | 196 (41.0) |
| ≥13 years | 177 (50.3) | 225 (47.1) |
| **Paternal occupation, n (%)** | | |
| Sick leave/Studying | 15 (4.2) | 20 (4.1) |
| Unemployed | 8 (2.2) | 4 (0.8) |
| Employed | 335 (93.6) | 459 (95.0) |

| | | |
|---|---|---|
| **Paternal annual salary (NOK), n (%)** | | |
| <200,000 | 29 (8.3) | 48 (10.3) |
| 200,000-399,999 | 196 (56.3) | 248 (53.2) |
| ≥400,000 | 123 (35.3) | 170 (36.5) |
| **Paternal type 1 diabetes, n (%)** | 7 (1.8) | 4 (0.8) |
| **Paternal celiac disease, n (%)** | 19 (5.0) | 0 (0.0) |
| **Paternal smoking in pregnancy, n (%)** | 60 (16.3) | 97 (19.6) |
| **Parental cohabitation, n (%)** | 356 (97.5) | 488 (98.4) |

Data from Norwegian Mother and Child Cohort (MoBa), the Norwegian Patient Register and the Medical Birth Registry of Norway. [A] HLA, human leukocyte antigen; DQ2.5: *HLA-DQA1*05:01-DQB1*02:01*, DQ2: DQ2.5 or DQ2.2; DQ2.2: *HLA-DQA1*02:01-DQB1*02:02;* DQ8: HLA-DQA1*03-DQB1*0302. [B] Genetic risk score based on 44 non-HLA risk alleles linked to celiac disease. IQR, interquartile range; NOK, Norwegian krone, the national currency of Norway.

***Supplementary Table 5.*** *Complete-case analysis on the association of cord blood cotinine concentration and childhood celiac disease.*

| | **Celiac disease** n=381 (%) | **Random controls** n=529 (%) | **Model I** | | **Model II** | | **Model III** | |
|---|---|---|---|---|---|---|---|---|
| | | | *aOR* | *95% CI* | *aOR* | *95% CI* | *aOR* | *95% CI* |
| **Trichotomous cotinine groups** ["Maternal exposure level"] | | | | | | | | |
| *Cotinine <1.0 nmol/L* ["No smoke exposure"] | 329 (86.4) | 433 (81.9) | | Ref. | | Ref. | | Ref. |
| *Cotinine 1.0-29.9 nmol/L* ["ETS/occasional smoking"] | 32 (8.4) | 49 (9.3) | 0.84 | 0.53-1.33 | 0.89 | 0.55-1.42 | 1.01 | 0.58-1.73 |
| *Cotinine ≥30.0 nmol/L* ["Daily smoking"] | 20 (5.2) | 47 (8.9) | 0.54 | 0.31-0.95 | 0.57 | 0.33-1.04 | 0.64 | 0.31-1.31 |
| **Per change in cotinine group** [A] | | | 0.76 | 0.59-0.97 | 0.78 | 0.60-1.02 | 0.85 | 0.62-1.15 |
| *Stratified by genetic risk* [B] | | | | | | | | |
| Moderate-risk HLAs, n=549 | | | 0.79 | 0.57-1.08 | 0.80 | 0.57-1.13 | 0.73 | 0.47-1.14 |
| High-risk HLAs, n=124 | | | 1.00 | 0.37-2.75 | 0.83 | 0.26-2.51 | 3.17 | 0.56-17.96 |

All analyses were adjusted for calendar year of birth and degree of hemolysis of cord blood samples (model I). Model II also included maternal education level, while Model III in addition to previous covariates also accounted for parental type 1 diabetes, celiac disease, income, occupation, cohabitation and paternal education level. The number of children varies between analyses because of missing data (model I: n=910; model II: n=856; model III: n=733). [A] Analysis of trend over categories of increasing cotinine concentrations. [B] Stratified analyses by the child's human leukocyte antigen, HLA, genotype classified as conferring a moderate and high risk for celiac disease. Analyses adjusted for non-HLA genetic risk score. See Methods for details. The low-risk HLA group was excluded due to lack of events. 95% CI, 95% confidence interval; aOR, adjusted odds ratio; ETS, environmental tobacco smoke.

## SUPPLEMENTARY FIGURE LEGENDS

***Supplementary Figure 1***. *Directed acyclic graph depicting the hypothesized relationships between maternal smoking in pregnancy and offspring celiac disease.*

Confounding variables are coloured in red and mediating variables in blue. Biasing paths and causal paths are coloured in red and green, respectively. Child's sex is shown in white indicating predictor for celiac disease that was included as an adjustment variable despite not being associated with maternal smoking. CD-associated HLA and non-HLA genetic risk markers are shown in grey indicating unmeasured predictor for celiac disease as they were only measured in the nested case-control study. To ease the interpretation of the diagram, arrows between predictors and mediators (e.g. the arrow between sex and birth weight) and arrows between confounders and mediators (e.g. arrow between parental celiac disease and birthweight) have been omitted because these do not have any consequence for the selection of adjustment variables. All analyses were adjusted for calendar year of birth (model I) and further adjusted for maternal education level (model II). In MoBa and our nested case-control sample we were also able to adjust for parental type 1 diabetes, CD, income, occupation, cohabitation and paternal education level (model III). A sensitivity analysis considered the effect of additional adjustment for parental age at delivery, maternal infections in pregnancy, the child's infection frequency by age six months, sex, breastfeeding duration, parity, delivery mode, gestational age and birth weight. In our nested case-control study we were also able to adjust for HLA and non-HLA genetic risk markers for CD.

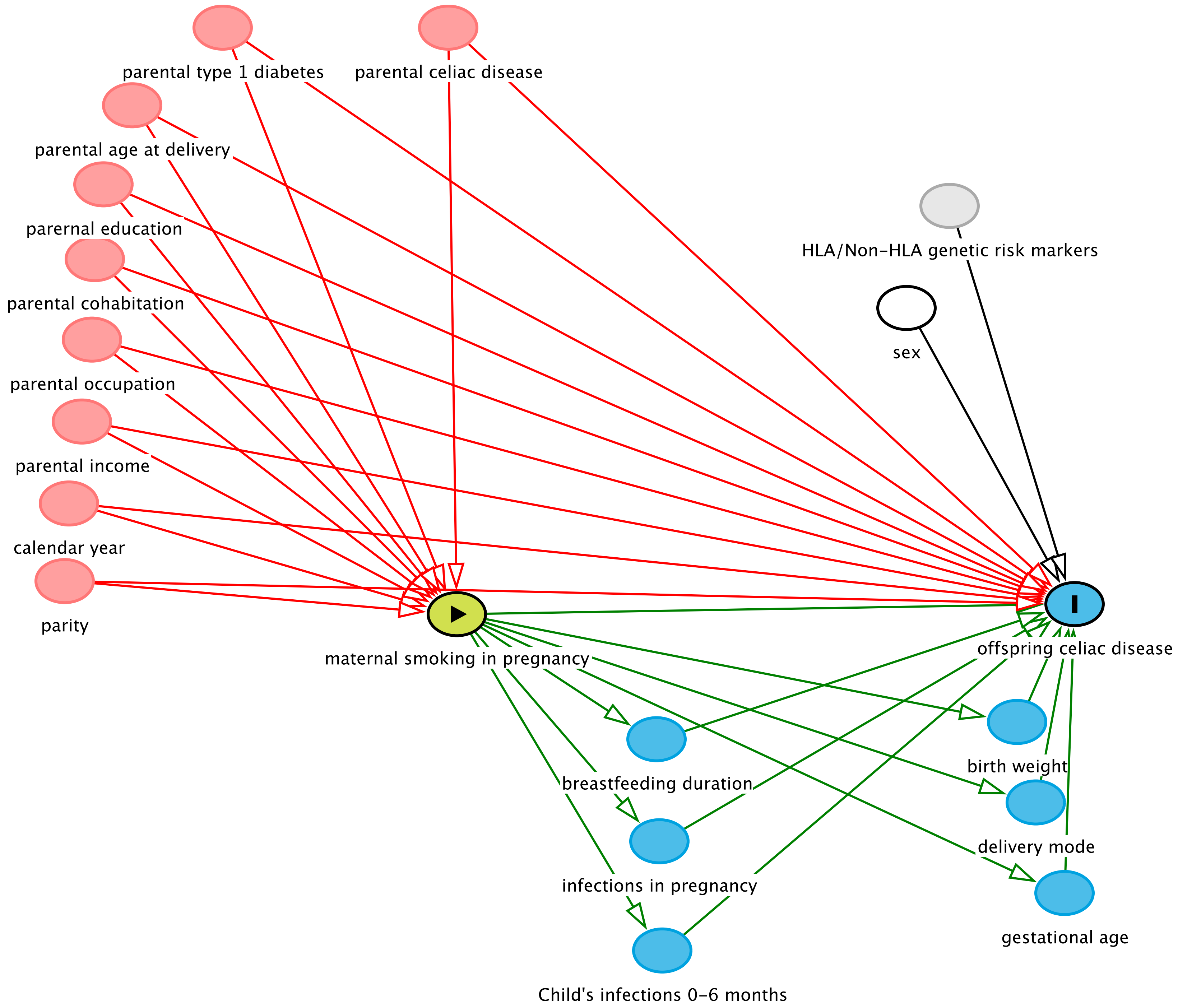

parental type 1 diabetes
parental celiac disease
parental age at delivery
parernal education
parental cohabitation
parental occupation
parental income
calendar year
parity
HLA/Non-HLA genetic risk markers
sex
maternal smoking in pregnancy
offspring celiac disease
breastfeeding duration
birth weight
infections in pregnancy
delivery mode
gestational age
Child's infections 0-6 months

***Supplementary Figure 2.*** *Complete-case analyses of the association of parental smoking with childhood celiac disease in the MoBa cohort.*

All analyses were adjusted for calendar year of birth (model I). Model II also included maternal education level, while Model III in addition to previous covariates also accounted for parental type 1 diabetes, celiac disease, income, occupation, cohabitation and paternal education level The number of children varies between analyses because of missing data. 95% CI, 95% confidence interval; aOR, adjusted odds ratio.

| | Celiac disease n=1035 (%) | No celiac disease n=92,984 (%) | Model I aOR (95% CI) | Model II aOR (95% CI) | Model III aOR (95% CI) |
|---|---|---|---|---|---|
| **MATERNAL SMOKING** | | | | | |
| **Ever smoked** | | | | | |
| Never-smoker | 558 (54.1) | 46,847 (50.8) | Ref. | Ref. | Ref. |
| Ever-smoker | 473 (45.9) | 45,431 (49.2) | 0.87 (0.77-0.99) | 0.89 (0.78-1.01) | 0.92 (0.80-1.06) |
| **Smoking 0-3 months before pregnancy** | | | | | |
| No smoking | 769 (74.6) | 66,593 (72.2) | Ref. | Ref. | Ref. |
| Smoking | 262 (25.4) | 25,589 (27.8) | 0.89 (0.77-1.02) | 0.90 (0.78-1.05) | 0.94 (0.79-1.10) |
| **Smoking in pregnancy** | | | | | |
| No smoking | 827 (80.1) | 71,546 (77.3) | Ref. | Ref. | Ref. |
| Smoking | 205 (19.9) | 20,966 (22.7) | 0.84 (0.72-0.99) | 0.86 (0.73-1.01) | 0.88 (0.73-1.05) |
| **Duration of smoking in pregnancy** | | | | | |
| No smoking | 848 (82.2) | 72,958 (79.0) | Ref. | Ref. | Ref. |
| Smoking <18 preg. weeks | 132 (12.8) | 12,050 (13.0) | 0.94 (0.78-1.13) | 0.94 (0.78-1.14) | 0.93 (0.75-1.15) |
| Smoking ≥18 preg. weeks | 52 (5.0) | 7401 (8.0) | 0.60 (0.45-0.80) | 0.62 (0.46-0.83) | 0.61 (0.43-0.86) |
| **Amount of daily smoking in pregnancy** | | | | | |
| *Per cigarette* | | | 0.92 (0.87-0.97) | 0.92 (0.88-0.98) | 0.92 (0.86-0.98) |
| 0 [no smoking] | 977 (94.4) | 84,789 (91.2) | Ref. | Ref. | Ref. |
| ≤2 cigarettes | 25 (2.4) | 3149 (3.4) | 0.69 (0.46-1.02) | 0.70 (0.47-1.04) | 0.63 (0.39-1.02) |
| >2 cigarettes | 33 (3.2) | 5020 (5.4) | 0.57 (0.40-0.81) | 0.59 (0.41-0.84) | 0.58 (0.38-0.89) |
| **Timing of smoking cessation** [explorative analysis] | | | | | |
| Never-smoker | 558 (67.5) | 46,847 (64.0) | Ref. | Ref. | Ref. |
| Cessation before pregnancy | 64 (7.7) | 5341 (7.3) | 1.01 (0.77-1.32) | 1.01 (0.77-1.32) | 1.11 (0.84-1.47) |
| Smoking <18 preg. weeks | 161 (19.5) | 14,374 (19.6) | 0.94 (0.78-1.12) | 0.94 (0.78-1.13) | 0.98 (0.80-1.20) |
| Smoking ≥18 preg. weeks | 44 (5.3) | 6592 (9.0) | 0.56 (0.41-0.76) | 0.57 (0.41-0.78) | 0.55 (0.38-0.81) |
| **PATERNAL SMOKING** | | | | | |
| **Smoking before pregnancy** | | | | | |
| No smoking | 771 (75.1) | 67,448 (73.2) | Ref. | Ref. | Ref. |
| Smoking | 256 (24.9) | 24,751 (26.8) | 0.90 (0.78-1.04) | 0.93 (0.80-1.07) | 1.00 (0.85-1.18) |
| **Smoking in pregnancy** | | | | | |
| No smoking | 857 (83.0) | 73,692 (79.7) | Ref. | Ref. | Ref. |
| Smoking | 175 (17.0) | 18,740 (20.3) | 0.80 (0.68-0.95) | 0.82 (0.69-0.97) | 0.86 (0.71-1.04) |
| **ENVIRONMENTAL TOBACCO SMOKE (ETS)** | | | | | |
| **Prenatal exposure to ETS** | | | | | |
| *Per daily hours of ETS* | | | 0.98 (0.96-0.99) | 0.98 (0.97-1.00) | 0.99 (0.97-1.00) |
| Not exposed | 915 (89.0) | 79,237 (86.4) | Ref. | Ref. | Ref. |
| Exposed | 113 (11.0) | 12,473 (13.6) | 0.78 (0.64-0.96) | 0.80 (0.65-0.98) | 0.87 (0.70-1.09) |
| **Postnatal exposure to ETS** | | | | | |
| Not exposed | 744 (81.7) | 63,144 (78.0) | Ref. | Ref. | Ref. |
| Moderately exposed | 80 (8.8) | 8299 (10.2) | 0.82 (0.64-1.04) | 0.82 (0.65-1.05) | 0.88 (0.68-1.13) |
| Highly exposed | 87 (9.5) | 9532 (11.8) | 0.78 (0.62-0.97) | 0.79 (0.63-0.99) | 0.84 (0.65-1.09) |

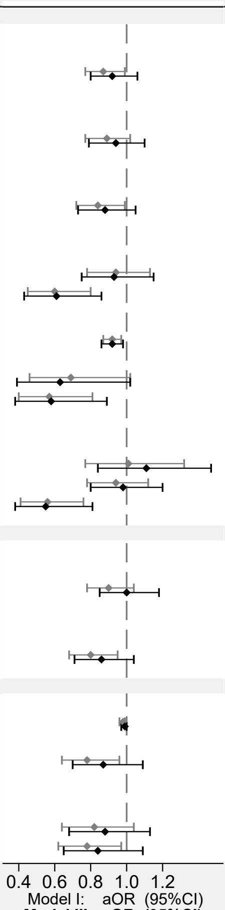

***Supplementary Figure 3.*** *Complete-case analysis on the association of maternal smoking in pregnancy recorded in the Medical Birth Registry of Norway and celiac disease diagnosis in offspring.*

Smoking recorded around 10 and 36 weeks of pregnancy. The primary analysis was based on a register-based cohort of Norwegian children born in 2004-2012. The explorative analysis examines the association among participants of the Norwegian Mother and Child (MoBa) cohort. Model I included calendar year of birth while model II was further adjusted for maternal education level, 95% CI, 95% confidence interval; aOR, adjusted odds ratio.

| **Register-based cohort:** | **Celiac disease** n=1570 (%) | **No celiac disease** n=440,723 (%) |
|---|---|---|
| **Duration of smoking in pregnancy** | | |
| No smoking | 1369 (87.2) | 383,438 (87.0) |
| Smoking only in early pregnancy | 82 (5.2) | 21,536 (4.9) |
| Smoking in early and late pregnancy | 119 (7.6) | 35,749 (8.1) |
| MoBa, explorative analysis: | Celiac disease n=855 (%) | No celiac disease n=77,397 (%) |
| **Duration of smoking in pregnancy** | | |
| No smoking | 792 (92.6) | 69,192 (89.4) |
| Smoking only in early pregnancy | 40 (4.7) | 4126 (5.3) |
| Smoking in early and late pregnancy | 23 (2.7) | 4079 (5.3) |

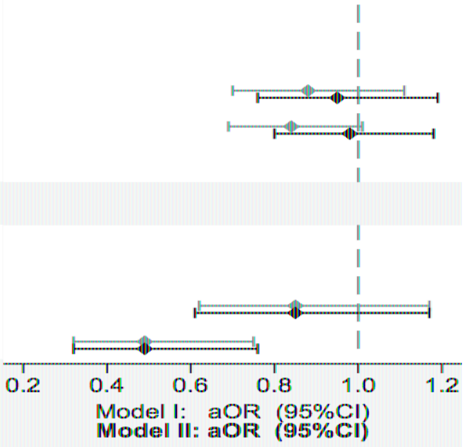

***Supplementary Figure 4.*** *Sensitivity analysis on the effect of additional adjustments of parental and child characteristics recorded in the MoBa cohort.*

Model III includes calendar year of birth, parental education level, type 1 diabetes, CD, income, occupation and cohabitation. 95% CI, 95% confidence interval; aOR, adjusted odds ratio.

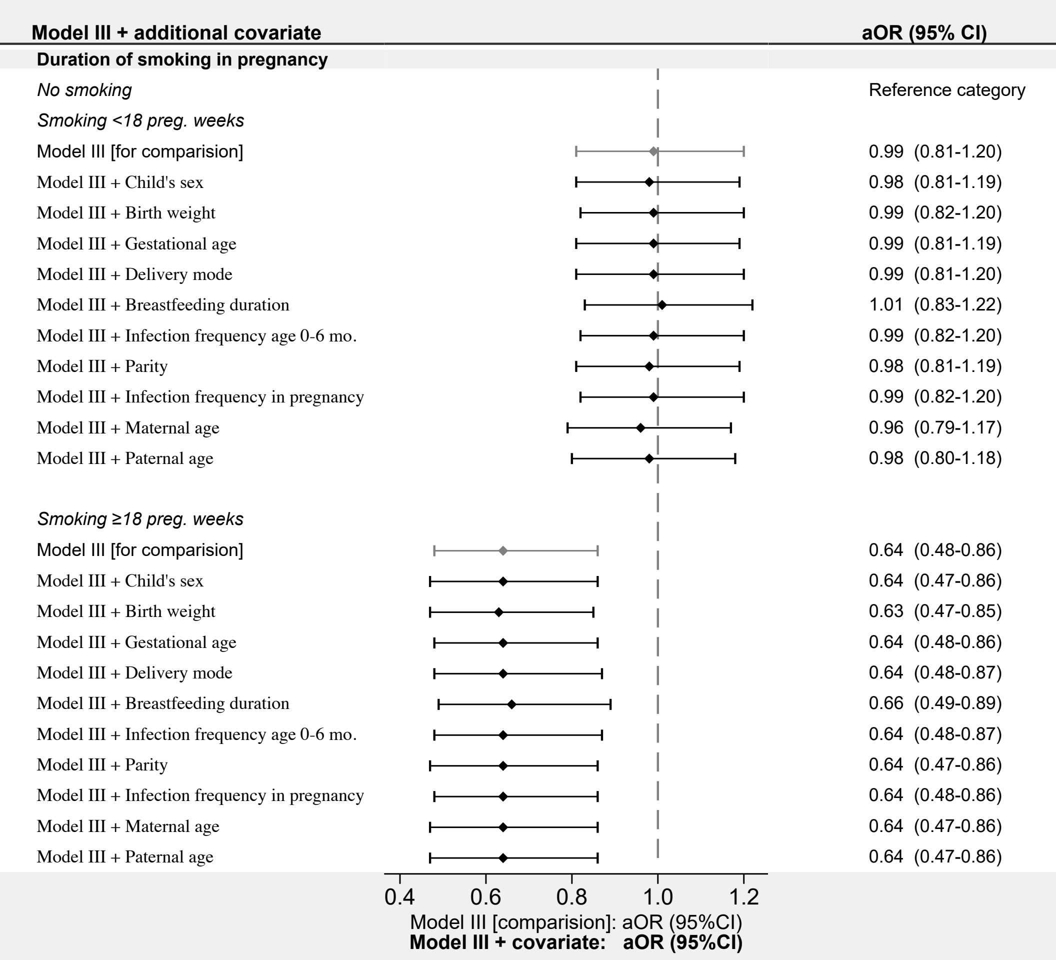
Model III + additional covariate
aOR (95% CI)
Duration of smoking in pregnancy
No smoking
Reference category
Smoking <18 preg. weeks
Model III [for comparision] 0.99 (0.81-1.20)
Model III + Child's sex 0.98 (0.81-1.19)
Model III + Birth weight 0.99 (0.82-1.20)
Model III + Gestational age 0.99 (0.81-1.19)
Model III + Delivery mode 0.99 (0.81-1.20)
Model III + Breastfeeding duration 1.01 (0.83-1.22)
Model III + Infection frequency age 0-6 mo. 0.99 (0.82-1.20)
Model III + Parity 0.98 (0.81-1.19)
Model III + Infection frequency in pregnancy 0.99 (0.82-1.20)
Model III + Maternal age 0.96 (0.79-1.17)
Model III + Paternal age 0.98 (0.80-1.18)
Smoking ≥18 preg. weeks
Model III [for comparision] 0.64 (0.48-0.86)
Model III + Child's sex 0.64 (0.47-0.86)
Model III + Birth weight 0.63 (0.47-0.85)
Model III + Gestational age 0.64 (0.48-0.86)
Model III + Delivery mode 0.64 (0.48-0.87)
Model III + Breastfeeding duration 0.66 (0.49-0.89)
Model III + Infection frequency age 0-6 mo. 0.64 (0.48-0.87)
Model III + Parity 0.64 (0.47-0.86)
Model III + Infection frequency in pregnancy 0.64 (0.48-0.86)
Model III + Maternal age 0.64 (0.47-0.86)
Model III + Paternal age 0.64 (0.47-0.86)
0.4
0.6
0.8
1.0
1.2
Model III [comparision]: aOR (95%CI)
Model III + covariate: aOR (95%CI)

## SUPPLEMENTARY METHODS

### *Recording of maternal smoking in the Medical Birth Registry of Norway*

The Medical Birth Registry of Norway (MBRN) is held by the Norwegian Institute of Health.[1] The registry contains information on essentially all births in Norway since 1967. Information on maternal smoking in pregnancy is available since 1999. Women may choose not to register smoking status in MBRN; in 16% of children born in 2004-2012 the mother declined to register smoking.

At the antenatal care clinics, the mother's current smoking status (or her declination for it to be registered) is documented in a medical record form ("Helsekort for gravide");[2] data are recorded at the time of the first antenatal visit (around 10 weeks of pregnancy, range 8-12 weeks) and at the last antenatal visit at 36 weeks of pregnancy. On both occasions, the mother's current smoking status is indicated by check boxes: "non smoker", "occasional smoker, not daily" or "daily smoker", and, if smoking daily, the number of cigarettes smoked per day.[3]

At the delivery units, maternal smoking status as recorded in the medical record form is transferred to a summarizing form sent to the MBRN, originally as paper forms that were computerized at the Norwegian Institute of Health. Since 2005, paper forms have been gradually replaced by an electronic transmission of information to the MBRN (by 2014, summarized paper forms were completely abandoned).[3] Minor differences exist between the recordings of maternal smoking in the paper-based and computer-based MBRN record systems;[3] while the original paper form recorded maternal smoking in "early" and "late" pregnancy, the electronic version requests maternal smoking in "first" and "last" trimester. Critically, however, both procedures defines "early"/"first trimester" as the mother's current smoking status as recorded at the time of the first antenatal visit (around 10 weeks of pregnancy) and "late"/"last trimester" as her current smoking status at the last antenatal visit at 36 weeks of pregnancy.

Since 1999, MBRN also provides information on maternal smoking before pregnancy (non-smoker, occasional and daily smoker). However, data on smoking during the pre-conception period suffer from a high degree of missingness (missing for 85% of children born in 2004-2012); for that reason, the current study did not examine smoking before pregnancy.

**References**

1. Irgens LM. The Medical Birth Registry of Norway. Epidemiological research and surveillance throughout 30 years. Acta Obstet Gynecol Scand 2000; 79:435-9.
2. Helsekort for gravide. 2018. Available from https://helsedirektoratet.no/retningslinjer/helsekort-for-gravide-kontinuasjonsark-veileder-og-plastlomme.
3. DATAKVALITET PÅ RØYKEDATA I MEDISINSK FØDSELSREGISTER. 2015. [Cited Folkehelseinstituttet, Avdeling for helseregistre.] Available from https://www.fhi.no/globalassets/dokumenterfiler/helseregistre/mfr/2016---mfr-roykedata-.pdf.

### *Details on genotyping of cord blood*

In our nested case-control analysis of cord blood cotinine we accounted for the child's human leukocyte antigen (HLA) genotype classified as conferring a high risk for celiac disease (CD) (homozygous for *DQA1*05:01-DQB1*02:01* [DQ2.5] or DQ2.5/*DQA1*02:01-DQB1*02:02* [DQ2.2]), moderate risk (DQ2.5/X or at least one of DQ2.2 or *DQA1*03-DQB1*03:02* [DQ8]) and a low risk (any other genotype).[1] As previously described,[2] we computed a non-HLA genetic risk score for CD defined as the child's sum of 44 risk alleles that previously have been linked to CD at genome

wide significance.[1, 3] Using DNA extracted from cord blood, genotyping was performed at the Core Facility for Genotyping at Oslo University Hospital (Oslo, Norway) on a Custom GoldenGate assay (Illumina, San Diego, CA). Genotypes for HLA were inferred from 66 tag single nucleotide polymorphisms using HLA:IMP*02 software[4] and thereafter confirmed through allele-specific amplification (Gillespie's Diabetes and Metabolism lab at School of Clinical Sciences, Bristol University, UK).

**References**


**1.** Trynka G, Hunt KA, Bockett NA, et al. Dense genotyping identifies and localizes multiple common and rare variant association signals in celiac disease. *Nat Genet.* Dec 2011;43(12):1193-1201.

**2.** Mårild K, Vistnes M, Tapia G, et al. Midpregnancy and cord blood immunologic biomarkers, HLA genotype, and pediatric celiac disease. *J Allergy Clin Immunol.* May 2017;139(5):1696-1698.

**3.** Romanos J, Rosen A, Kumar V, et al. Improving coeliac disease risk prediction by testing non-HLA variants additional to HLA variants. *Gut.* Mar 2014;63(3):415-422.

**4.** Dilthey A, Leslie S, Moutsianas L, et al. Multi-population classical HLA type imputation. *PLoS Comput Biol.* 2013;9(2):e1002877.

***Excerpts from MoBa-questionnaires used to assess parental smoking***

The complete questionnaires are available at www.fhi.no/moba.

**Questionnaire administered at pregnancy week 18 (questions 87-96, 100, 104)**

12

## Living habits

87. Did your mother smoke when she was pregnant with you? AA1348
No
Yes
Don't Know

88. Are you exposed to passive smoking at home? AA1349
No Yes

89. If yes, how many hours a day are you exposed to passive smoking? AA1350
hours per day

104: responses in questionnaire1b.doc and the Excel file don't match

90. Are you exposed to passive smoking at work? AA1351
No Yes

91. If yes, how many hours a day are you exposed to passive smoking? AA1352
hours per day

92. Did the baby's father smoke before you became pregnant? AA1353
No Yes

93. Does he smoke now? AA1354
No
Yes

94. Have you ever smoked? AA1355
No (proceed to question 104)
Yes

95. Do you smoke now (after you became pregnant)?
No AA1356
Sometimes AA1357 cigarettes per week
Daily AA1358 cigarettes per day

96. Did you smoke during the last 3 months before you became pregnant this time?
No AA1359
Sometimes AA1360 cigarettes per week
Daily AA1361 cigarettes per day

97. How old were you when you started to smoke on a daily basis?
AA1362 Years

98. Have you stopped smoking completely?
No Yes AA1363

99. If yes, how old were you when you stopped smoking?
AA1364 Years

100. If you stopped smoking after you became pregnant, in which week of pregnancy did you stop?
AA1365 week of pregnancy

101. How long after you get up in the morning until you light your first cigarette? AA1366
5 minutes
6-29 minutes
30-60 minutes
More than one hour

102. Do you smoke when you are ill? AA1367
No Yes

103. Do you smoke more often during the first few hours after you wake up than you do during the rest of the day? AA1368
No Yes

104. If you have used other kinds of nicotine indicate which and when you used them.

| | Before pregnancy | During pregnancy |
|---|---|---|
| Chewing tobacco/snuff | AA1369 | AA1370 |
| Nicotine chewing gum | AA1371 | AA1372 |
| Nicotine adhesive patch | AA1373 | AA1374 |
| Nicotine inhaler | AA1375 | AA1376 |

105. What was your fluid consumption (number of cups/glasses) per day before and during pregnancy? (1 mug = 2 cups, 1 small plastic bottle (0.5 litre) = 4 cups, 1 large plastic bottle (1.5 litres) = 12 cups)

| | Number of cups/glasses: Before pregnancy | Now | Decaffeinated (Enter a cross) |
|---|---|---|---|
| 1 Filter coffee | AA1377 | AA1378 | AA1379 |
| 2 Instant coffee | AA1380 | AA1381 | AA1382 |
| 3 Boiled coffee | AA1383 | AA1384 | AA1385 |
| 4 Tea | AA1386 | AA1387 | AA1388 |
| 5 Herbal tea | AA1389 | AA1390 | AA1391 |
| 6 Coca Cola/Pepsi etc. | AA1392 | AA1393 | AA1394 |
| 7 Other fizzy drinks | AA1395 | AA1396 | AA1397 |
| 8 Diet Coca Cola/Pepsi | AA1398 | AA1399 | AA1400 |
| 9 Other diet fizzy drinks | AA1401 | AA1402 | AA1403 |
| 10 Tap water | AA1404 | AA1405 | |
| 11 Bottled water | AA1406 | AA1407 | |

| | Before pregnancy | Now | Ecological (Enter a cross) |
|---|---|---|---|
| 12 Juice/squash | AA1408 | AA1409 | AA1410 |
| 13 Diet juice/squash | AA1411 | AA1412 | AA1413 |
| 14 Milk (skim, low fat, whole) | AA1414 | AA1415 | AA1416 |
| 15 Yogurt, all types | AA1417 | AA1418 | AA1419 |
| 16 Yogurt/active Lactobacillus | AA1420 | AA1421 | AA1422 |
| 17 Other type of cultured milk - Kefir | AA1423 | AA1424 | AA1425 |
| 18 Other | AA1426 | AA1427 | AA1428 |

# Questionnaire administered at around pregnancy week 30 (questions 98-102)



**91. If yes, which animals have you come into contact with and how often?**

| | Daily | 3–6 times a week | 1-2 times a week | Less often |
|---|---|---|---|---|
| Dog | CC1018 | | | |
| Cat | CC1019 | | | |
| Guinea pig/hamster/rabbit/rat, etc. | CC1020 | | | |
| Canary or other caged birds | CC1021 | | | |
| Hens and other poultry | CC1022 | | | |
| Cow/sheep/goat | CC1023 | | | |
| Horse | CC1024 | | | |
| Pig | CC1025 | | | |
| Other | CC1026 | | | |

**92. How many hours a day do you usually sleep now when you are pregnant?** CC1027

Over 10 hours
8–9 hours
6-7 hours
4-5 hours
Less than 4 hours

**93. Do you currently sleep on a waterbed or use an electric blanket?**

| | Yes | No |
|---|---|---|
| Waterbed | CC1028 | |
| Electric blanket | CC1029 | |

**94. Can you rest during the day (both at home and at work)?** CC1030

No
Yes

**95. Have you been in a sauna while you have been pregnant?** CC1031

No
1–5 times
6-10 times
More than 10 times

**96. Have you been in a solarium while you have been pregnant?** CC1032

No
1-5 times
6-10 times
More than 10 times

**97. Are you exposed to passive smoking either at home or at work? If yes, how many hours a day?**

| | No | Yes | No. of hrs |
|---|---|---|---|
| Home | CC1033 | | CC1034 |
| Work | CC1035 | | CC1036 |

**98. Do you smoke at present? If yes, how many cigarettes?**

No CC1037
Sometimes CC1038 Cigarettes per week
Daily CC1039 Cigarettes per day

**99. Does the baby's father smoke at present? If yes, how many cigarettes?**

No CC1040
Sometimes CC1041 Cigarettes per week
Daily CC1042 Cigarettes per day

**100. If one or both of you have stopped smoking during the pregnancy, in which week of pregnancy did you stop?**

You CC1043 CC1044 Week of pregnancy
Baby's father CC1045 CC1046 Week of pregnancy

**101. If you or the baby's father have smoked during the pregnancy, were there periods during which you or the baby's father did not smoke?** *(Fill in the weeks during pregnancy when you did not smoke.)*

Weeks of pregnancy without smoking

| | 0–4 | 5–8 | 9–12 | 13–16 | 17–20 | 21–24 | 25–28 | 29+ |
|---|---|---|---|---|---|---|---|---|
| You | CC1047 | CC1048 | CC1049 | CC1050 | CC1051 | CC1052 | CC1053 | CC1054 |
| Baby's father | CC1055 | CC1056 | CC1057 | CC1058 | CC1059 | CC1060 | CC1061 | CC1062 |

**102. Have you used other forms of nicotine after the 13th week of pregnancy?**

| | No | Yes |
|---|---|---|
| Nicotine chewing gum | CC1063 | |
| Nicotine patches | CC1064 | |
| Nicotine inhaler | CC1065 | |
| Chewing tobacco/snuff | CC1066 | |

**103. Have you used any of the following substances after the 13th week of pregnancy?**

| | No | Yes |
|---|---|---|
| Hash | CC1067 | |
| Amphetamine | CC1068 | |
| Ecstasy | CC1069 | |
| Cocaine | CC1070 | |
| Heroin | CC1071 | |

**104. Have you ever used any of the following substances?** (Fill in for each item.)

| | No | Previously | Last 6 months before pregnancy | During this pregnancy |
|---|---|---|---|---|
| Anabolic steroids | CC1072 | | | |
| Testosterone products | CC1073 | | | |
| Growth hormones *(e.g. genotropin/somatropin)* | CC1074 | | | |

## Questionnaire administered at child's age 6 months (question 81)



**79. How often are you physically active at present?** *(Enter a cross in a box for each item.)*

| | Never | 1-3 times amonth | Once a week | Twice a week | Three times or more a week |
|---|---|---|---|---|---|
| 1 Didn't smoke | DD716 | | | | |
| 2 Brisk walking | DD717 | | | | |
| 3 Running/jogging/orienteering | DD718 | | | | |
| 4 Cycling | DD719 | | | | |
| 5 Training studio/weight training | DD720 | | | | |
| 6 Special gymnastics/aerobics for pregnant women | DD721 | | | | |
| 7 Aerobics/gymnastics/dancing without running and jumping | DD722 | | | | |
| 8 Aerobics/gymnastics/dancing with running and jumping | DD723 | | | | |
| 9 Dancing (swing, rock, folk) | DD724 | | | | |
| 10 Skiing | DD725 | | | | |
| 11 Ball sport | DD726 | | | | |
| 12 Swimming | DD727 | | | | |
| 13 Riding | DD728 | | | | |
| 14 Other | DD729 | | | | |

**80. Currently how often are you physically active (during your spare time or at work) that you get out of breath or sweat?**

| | Spare time | At work |
|---|---|---|
| Never | DD730 | DD731 |
| Less than once a week | | |
| Once a week | | |
| Twice a week | | |
| 3-4 times a week | | |
| 5 times or more a week | | |

**81. What were your and your partner/husband's smoking habits during the last 3 months of your pregnancy and in the period after the birth?** *(Enter a cross in a box for each period.)*

| | Yourself | | | Your partner/husband | | |
|---|---|---|---|---|---|---|
| | Last 3 mths during pregnancy | 0-3 mths after birth | 4-6 mths after birth | Last 3 mths during pregnancy | 0-3 mths after birth | 4-6 mths after birth |
| Didn't smoke | | | | | | |
| Smoked sometimes | DD732 | DD733 | DD734 | DD735 | DD736 | DD737 |
| Smoked every day | | | | | | |
| If every day, number of cigarettes per day | DD738 | DD739 | DD740 | DD741 | DD742 | DD743 |
| If sometimes, number of cigarettes per week | DD1114 | DD1115 | DD1116 | DD1117 | DD1118 | DD1119 |

**82. Is your child ever present in a room where someone smokes?**

| | |
|---|---|
| No | DD744 |
| Yes, sometimes | |
| Yes, several times a week | |
| Yes, every day | |
| If every day, number of hours | DD745 |

**83. Did you take any of the following substances during the last 3 months of your pregnancy and after the birth?**
*(Enter a cross in a box for each item.)*

| | No | Yes, last 3 month of pregnancy | Yes after birth |
|---|---|---|---|
| Hanish | DD746 | DD747 | DD748 |
| Amphetamines | DD749 | DD750 | DD751 |
| Ecstasy | DD752 | DD753 | DD754 |
| Cocaine | DD755 | DD756 | DD757 |
| Heroin | DD758 | DD759 | DD760 |
| Other, specify: DD905 DD764 | DD761 | DD762 | DD763 |
